# Understanding the anticorrosive protective mechanisms of modified epoxy coatings with improved barrier, active and self-healing functionalities: EIS and spectroscopic techniques

**DOI:** 10.1038/s41598-017-15845-0

**Published:** 2017-11-15

**Authors:** Demian I. Njoku, Miaomiao Cui, Haigang Xiao, Baihui Shang, Ying Li

**Affiliations:** 1 Laboratory for Corrosion and Protection, Institute of Metal Research, Chinese Academy of Sciences, 62 Wencui Road, 110016 Shenyang, Liaoning, China; 2 University of Chinese Academy of Sciences (UCAS), 19A Yuquan Rd, Shijingshan District, Beijing, P. R. China 100049

## Abstract

The present investigation adopted long-term *in-situ* electrochemical and spectroscopic approaches to study the combined active, self-healing and passive protective mechanisms of a new class of innovative anti-corrosive coatings based on epoxy doped with clay nanotubes impregnated with active species for the protection of carbon steel in 3.5% NaCl solution. The suitability of the as-received clay nanotubes to encapsulate the active agents was confirmed by different spectroscopic measurements. Tube end stopper with Ferric ion and polymer encapsulation with chitosan cross-linked with glutaraldehyde were adopted to tunnel the release of the active agents loaded into the nanotubes. The improved passive barrier performances of the various innovative coatings were revealed by the electrochemical impedance spectroscopic, while their active feedback and self-healing abilities were revealed by the optical and spectroscopic techniques. The optical/spectroscopic techniques revealed the degree of pit formation at the steel/coating interface and the iron rust formation around the artificially marked defects, including the ability of the marked defects to self-heal over exposure times. Adhesion and impacts tests were adopted to compare the physical/mechanical properties of the various coatings. The results afforded insights into the effects of exposure time on the protective and failure behaviours of both the reference and modified coatings.

## Introduction

The use of organic protective coatings is one of the most effective methods to provide a direct barrier layer separating the underneath metal from the corrosive species in the external environment^1–4^. However, over lengthened exposure periods in aggressive environments, especially in the presence of defects or voids (often caused by the processes of coating application; transportation, installation or service), organic coating’s barrier integrity can be greatly altered leading to unexpected materials failures. Consequently, in recent times, research interests are focused on the development of new class of protective coatings that possess multiple protective functionalities such as passive (i.e. barrier against corrosive species and high adhesion to the metal surface), active (i.e. carriers of sacrificial pigments and/or corrosion inhibiting agents)^4–8^ and self-healing (presence of self-healing agents able to close-up the defective zones). Previously, active feedback functionality in protective coatings was achieved by the direct addition of inhibitive agents into the coatings matrix which is released upon a breach in the coating to mitigate corrosion process^4^. In fact, active systems are also beneficial to coatings without defects, since water and other corrosive ions can diffuse over time to the coating/steel interface and initiate undercoat corrosion. However, it has been extensively reported that a direct addition of active agents into a coating matrix is not efficient due to poor distribution, incompatibility and high leaching rates of most active agents^9‒10^. Thus, nowadays, the promising approach for impacting effective and sustainable active feedback functionality in protective coatings is through the addition of encapsulated active species into the coating formulation^2,10‒12^. This approach advances numerous benefits over direct additions such as (a) eliminates the undesirable interaction of active agents with the coating constituents, (b) enhances the even distribution of low soluble active agents in the coating matrix, and (c) reduces the leaching rate of high soluble active agents in aqueous systems^9–13^. Consequently, in this regard, several types of nano / micro containers have been introduced for the encapsulation of active agents, such as the inorganic non-metallic oxides (SiO_2_, silica particles, clays)^14–16^, metallic oxides (Mn_2_O_3_, CeO_2_)^17,18^, carbon nanoparticles (carbon nanotubes)^19–21^, and organic nano / micro-containers (polyelectrolytes, polymer shells)^16,22–24^. Regrettably, the industrial application of some of these nano/micro containers is confronted by some setbacks. For instance, the metallic nanoparticles and carbon nanotubes are very expensive coupled with the low loading efficiency of active agents. Also, polymer shells such as poly urea-formaldehyde and polyelectrolytes shells are both expensive and soft; thus, can easily rupture by the shear forces often encountered during the mixing of the resin. In recent times, one attractive alternative to the aforementioned containers is the use of halloysite clay nanotubes (HNTs). HNTs are abundantly available, relatively cheap and are compatible with many polymer coatings such as epoxy, acrylic, polyurethane etc^25–27^. The nano-thickness of HNTs has been reported to improve the barrier and mechanical properties of epoxy and other polymeric composite coatings^10,26–29^. Furthermore, HNTs possess internal lumen and interspaces for sufficient loading of active agents up to 10–20% by weight^11,12,16^, and have been used to encapsulate numerous chemical agents such as drugs, antifouling agents, inhibitors etc.^6,10,11,30,31^. In the present report, we demonstrate the ability to introduce additional active feedback and enhanced self-healing protective functionalities to the epoxy coating without sacrificing the inherent barrier and physical properties (rather improved them). The active species of choice are 2-mercaptobenzothiazole (MBT) and benzotriazole (BTA) - which are among the non-toxic and cost-effective triazole-based organic corrosion inhibitors that have demonstrated excellent corrosion inhibition abilities on different metals in different media^32–36^. Furthermore, ferric ions and cross-linked chitosan polymer were used to control the release rate of the active agents and to promote the self-healing property of epoxy. To better understand the protective mechanisms of our innovative coatings, and to provide evidence for potential application, long-time impedance spectroscopy technique (EIS) supported with scanning electron spectroscopic technique (SEM) were adopted. These complementary techniques were selected based on the fact that, previously, most active and self-healing protective coatings researchers employed a short-term electrochemical test methods^2,9–18^, which is not sufficient enough to predict the possible long-term protective ability of the coatings when deployed in service; and does not provide mechanistic insights into the long-term combined active, passive and self-healing protective actions. In this regard, we approximated the experimental conditions to real field situation by applying a coating thickness of ~200μm and extending the experimental time till significant weak protection is recorded. The impedance data revealed the improved passive barrier property of the modified coatings (compared with the un-modified epoxy coating), while optical and spectroscopic data revealed their self-healing and active feedback anti-corrosive properties. Physical tests were carried out to compare some physical/mechanical properties of both the modified and the reference coatings to evaluate the effects (if any) of doping active species laded HNTs (with and without surface treatments) in epoxy coating matrix.

## Results and Discussion

### Characterization of the as-received halloysite clay nanotubes sample

The XRD intensity pattern presented in the supporting information (Fig. SI1a) for the as-received halloysite clay nanoparticles followed the pattern for a halloysite 7 A with tiny peak for the hydrated 10 A form, which indicates that the as-received halloysite nanotubes used in this study composed mostly of the dehydrated 7 Å form with small amounts of 10 Å form^37,38^. The basal spacing reflection showed a sharp d_001_ peak at 12.3^0^ in 2Theta, which according to the Bragg’s law corresponds to a basal spacing of 0.72 nm which is typical of halloysite 7 A samples. In the same supporting material (Fig. SS1b and Table SS1), the FTIR absorption peaks and the corresponding assignments for the as-received samples are presented. The data collectively show that the as-received HNTs composed mainly: Al, Si, O and H elements which are consistent with the elemental compositions of halloysite nanoparticles with little or no Fe content^37–39^.

**Figure 1:**
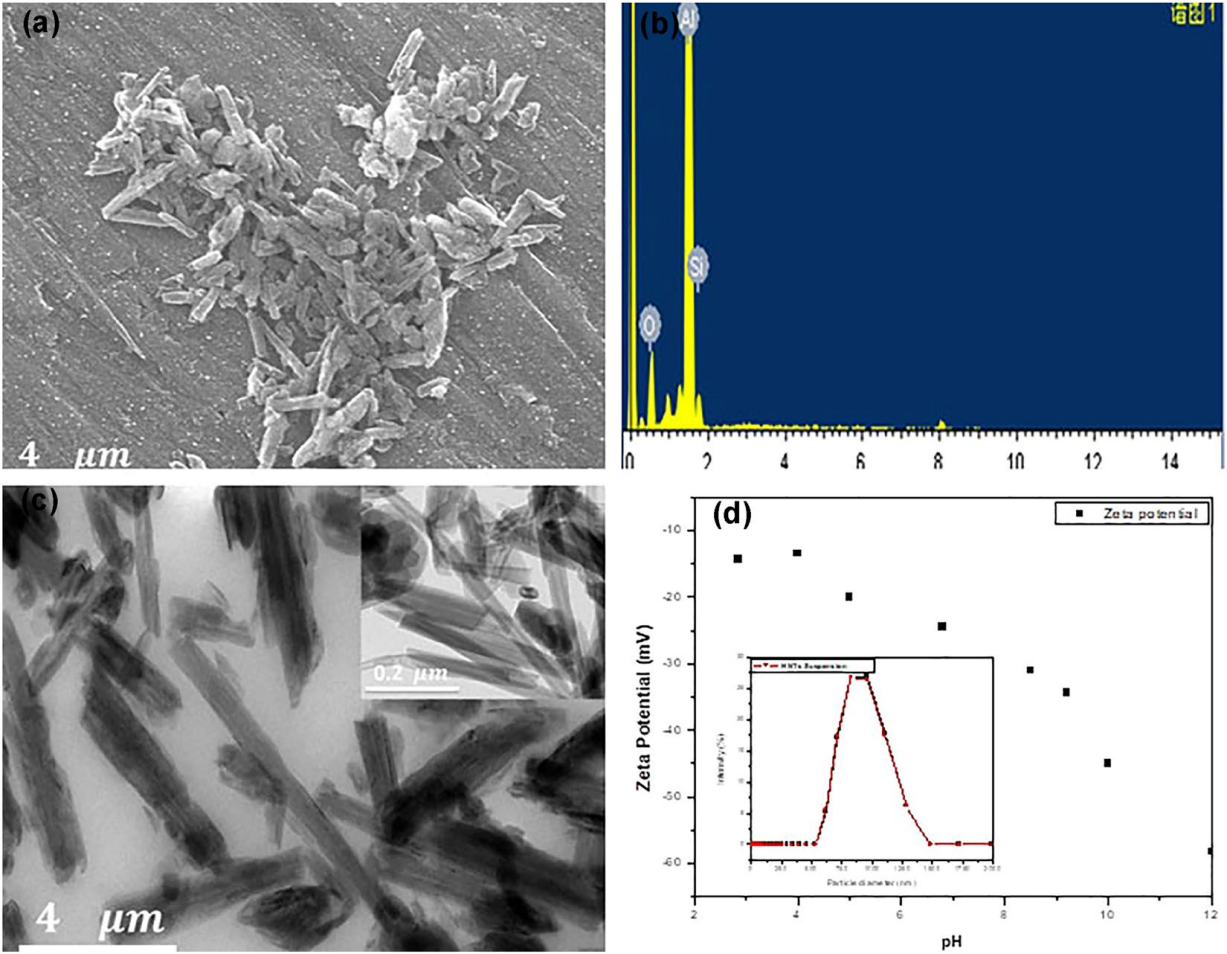
(**a**) SEM image; (**b**) EDS spectrum;(**c**) TEM image; (**d**) Zeta potential plot with particle size distribution data (inset); for the as-received halloysite clay nanotubes.

To evaluate the tubular morphology, composition, size and charge distribution of the as-received HNTs sample, SEM, EDS, TEM, particle size and Zeta-potential measurements were carried out on the sample. The SEM and TEM images for the as-received HNTs samples presented in Fig. 1a and c revealed that the nanoparticles are long and tubular; with internal lumen diameters of 15–25 nm (inset diagram–Fig. 1c), external diameters of 0.07–0.12 μm and lengths of 0.4–1.26 m. The EDS results in Fig. 1b showed defined peaks for Al, Si and O supporting the FTIR observation.

From the particle size analysis plot Fig. 1d (inset), the average size distribution of the sample (Z–average – d.nm) was 0.8–1 μm, which corresponds with the TEM result for the tube average diameters. The zeta potential plot shown in Fig. 1d indicated that the surface charge of the as–received HNTs sample was negative throughout the studied pH range (2–12). The negative surface charge of HNTs is related to the presence of Si–O (which is an acidic oxide) on the outermost surface of the nanoparticles; meanwhile, the inner surface contains Al–O which is amphoteric and can assume positive charge. According to Lewis and Deasy^30^, in aqueous solution, water causes the ionization of the outer surface Si–O (Si_surface_–OH → Si_surface_–O^−^ + OH^−^) thereby impacting negative charge on the surface of the HNTs as evidenced in the zeta potential curve. Hence, the various characterization results of the as-received HNTs confirmed that the nanotubes should readily serve as an excellent candidate for the encapsulation of the active agents with surface features permitting the binding of cationic polymers from solution (polymer encapsulation).

### Characterization of HNTs loaded with inhibiting compounds

The efficacy of our loading approach was evaluated with TGA–DTG, FTIR and XRD techniques. The TGA–DTG curves for the as-received halloysite clay nanotubes (Fig. SI2a) showed weight loss of ~1% during the initial stage of the heating process corresponding to the DTG inflection point at ~50 °C which can be ascribed to the dehydration of the particles with 10 Å basal spacing and/or the removal of the physically adsorbed water molecules^27,37^. The pronounced weight loss around 400–500 °C, corresponding to DTG inflection point around 480 °C can be ascribed to the removal of interlayer water or the dehydration of structural aluminol groups present in HNTs^27,37^. The TGA–DTG curves for the BTA and MBT loaded HNTs nanoparticles (Fig. SI2b,c) show additional weight losses around 150–200 °C corresponding to DTG inflection points around 180–250 °C. The TGA mass loss and the DTG inflection point at ~193 °C for HNTs-loaded with BTA are due to the evaporation of BTA^40^, whereas, the TGA mass loss and the corresponding DTG inflection point at ~219 °C have been previously related to the evaporation of an MBT derivative^41^. Furthermore, the presence of loaded inhibitor compounds in HNTs can be verified from the FTIR spectra presented in Fig. SI3a,c. The FTIR spectra show the presence of some peaks associated with the inhibitors (BTA and MBT) along with the peaks for HNTs. Interestingly, the XRD patterns for the BTA and MBT loaded HNTs nanoparticles presented in Fig. SS4a,b did not show any appreciable change in the 0.72 nm basal spacing, indicating that there were no intercalations at the interlayers of HNTs; and the inhibitors were probably loaded into the lumen and pores of the HNTs nanotubes. Thus, the loaded inhibitor crystals could be contained in the HNTs lumen for longer periods until it comes in contact with water or other corrosive electrolytes which can initiate its dissolution and subsequent release into the defective zones as free-moving corrosion inhibiting agents.

### Release kinetics

**Figure 2:**
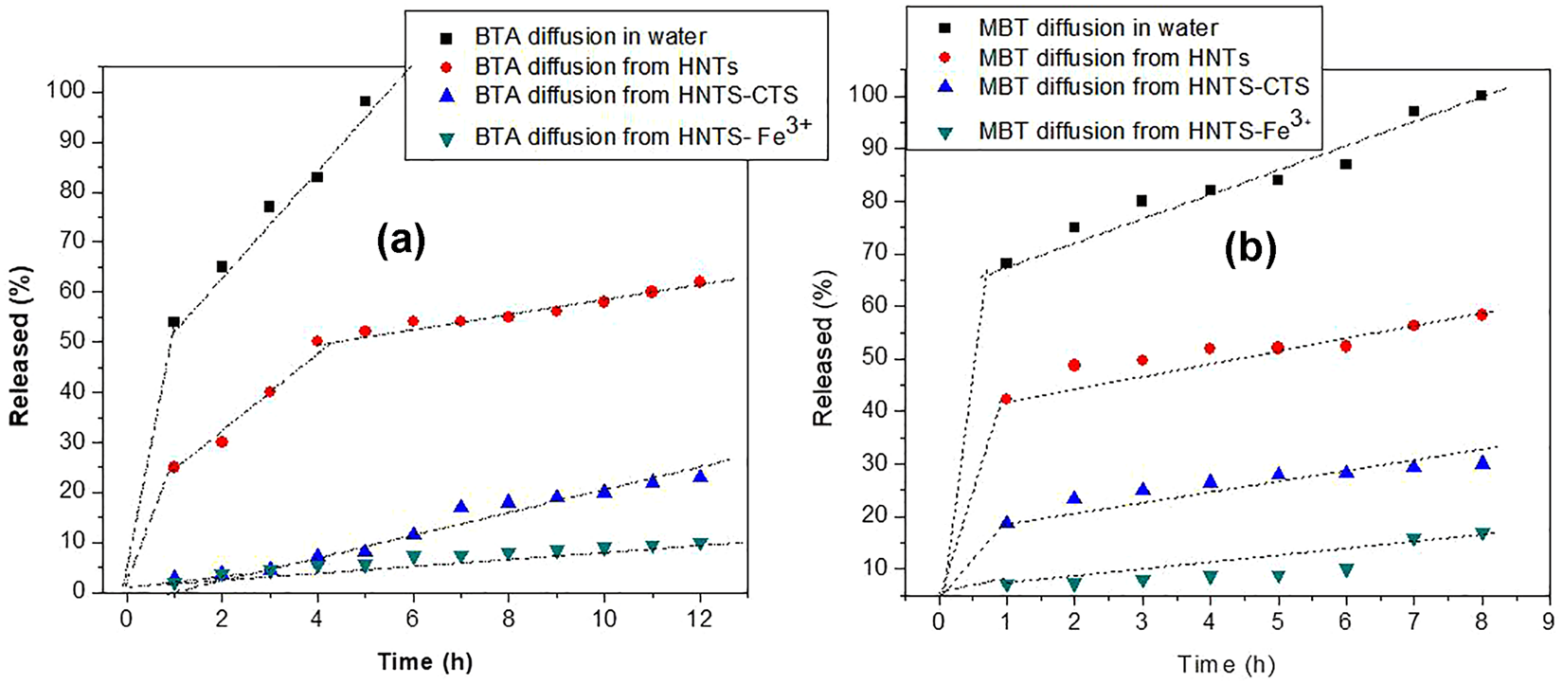
The diffusion profiles for BTA (**a**) and MBT (**b**): for the naked inhibitor crystals in water; inhibitor diffusion from loaded HNTs into water and inhibitor diffusion from loaded HNTs with interfacial treatments (Fe tube end capping and CTS tubular encapsulation) into water.

The profiles comparing the cumulative fraction of the diffusion of BTA and MBT crystals in water and their corresponding release from HNTs pores/lumen over time are presented in Fig. 2(a,b) respectively. Examination of the various profiles shows lower diffusion rate for the inhibitors loaded inside HNTs when compared with the diffusion of their crystals in water over time. Initial burst release of the inhibitors loaded in the HNTs was noticed for the first 1 h hour corresponding to 25–40% of total released loaded inhibitor compounds. This observation can be attributed to the release of the exogenously adsorbed inhibitor molecules propelled by the initial large concentration gradient in the system. The release profile of BTA from HNTs followed further two stages. The first stage could be attributed to the fast release of the BTA loaded in the external pores and the outermost part of the lumen (corresponding to a cumulative release of 60%); while the second stage corresponds to the release of inhibitor crystals loaded right inside the HNTs tube lumen. The slower release of inhibitors loaded inside the particles’ lumen can be related to the increased viscosity of the water in the lumen due to its interaction with the inner tube surface^36^. However, the release of MBT approximately followed only one-stage release profile after the first initial burst release. This shows that MBT crystallizes more inside the lumen and/or releases slowly due to its low solubility in water.

### Control release of inhibitive agents

**Figure 3:**
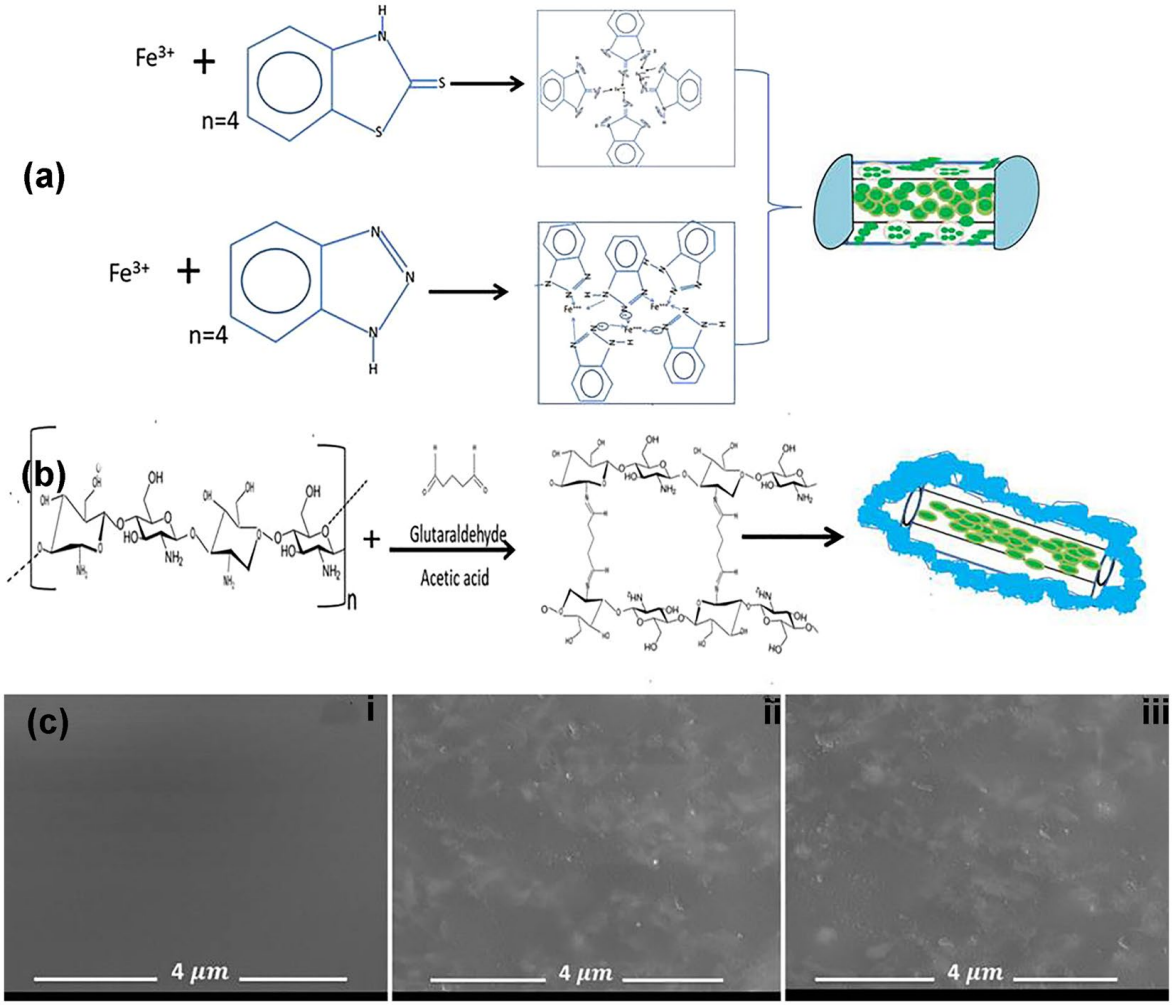
(**a**) Mechanism for Fe^3+^ tube ends stopper formation with BTA and MBT loaded HNTs, (**b**) mechanism for chitosan–glutaraldehyde (CTS) network HNTs tubular encapsulation, (**c**) SEM micro-morphologies for: epoxy coating (i); epoxy embedded with 5% loaded HNTs (ii); and epoxy coating embedded with 10% loaded HNTs (iii) – displaying the dispersed loaded HNTs nanotubes in the epoxy coating matrix.

It is necessary to control the release rate (including mitigating the unnecessary initial burst release) of the inhibiting species loaded in HNTs nanotubes so as to lengthen the release process time when exposed to wet systems. Tube end stopper (metal capping) achieved with Fe^3+^ and tubular encapsulation achieved with chitosan cross-linked with glutaraldehyde was used to control the release of the loaded active agents. In un-capped loaded HNTs systems, the active species simply diffuse out of the HNTs nanocontainers from the openings, whereas, in the presence of Fe^3+^, the diffusion rate of the active species will be delayed by the formed complex film on the outer openings of the lumen/pores as demonstrated in the Fig. 3a. Secondly, the formation of polymer hydrogel film on the surface of HNTs was achieved by the cross-linking of chitosan with glutaraldehyde. Since the surface of HNTs is negatively charged as discussed before, and chitosan is positively charged due to the presence of amine group^42,43^, the electrostatic attraction between the positively charged units would lead to a spontaneous adsorption of chitosan polymer film on HNTs surface and the film was thereafter stabilized by cross-linking with glutaraldehyde (CTS). The mechanism of tubular encapsulation by CTS has been illustrated in Fig. 3b. The capabilities of Fe^3+^ tube end stopper and CTS polymer encapsulation in delaying the release rates of the active species loaded in the HNTs nanotubes, including the mitigation of the initial burst release of the inhibiting agents can be verified from the various release profiles presented in Fig. 2a,b.

### Modelling the diffusion/release profiles

The diffusion of the inhibitor crystals (BTA and MBT) and their corresponding release from the HNTs lumen/pores in water were appropriately fitted to the Peppas model^24^,

1 $$\frac{{M}_{t}}{{M}_{\infty }}=K{t}^{n}$$

where M_t_ is the amount of the inhibitor compounds released at time (t), *M*_*∞*_ is the amount of inhibitor compounds released at the end of the release process (normalized as 100%), the parameter n is the release exponent - indicative of the characteristics of the release mechanism, and K is the release rate constant^24,44,45^. The corresponding fitting parameters (n and K) comparing the diffusion of the naked inhibitor crystals in water and their corresponding release from HNTs nanotubes are listed in Table SI2. Higher values of diffusion coefficients (K) were obtained for the diffusion of naked inhibitor crystals in water when compared with the corresponding diffusion coefficients for the inhibitor compounds loaded in HNTs. Going further, Weibull’s model was adopted to approximate the dissolution/release process times. Weibull expresses the accumulated fraction of the diffused/released species into the solution (M), at a time (t) by:

2 $$M=1-exp[\frac{-\,{(t-{T}_{i})}^{b}}{a}]\,$$

where *a* represents the time of the release process, T_i_, represents the lag time before the onset of the dissolution / release process (which is often taken to be zero), b, describes the shape of the dissolution profiles and can be classified as either: Case 1 – when the exponent b = 1 and the profile corresponds exactly to the shape of an exponential; Case 2 – when b> 1 and the profile has a sigmoid, s-shaped followed by a turning point; Case 3 – when b< 1 and the profile has an initial high slope followed by a consistent part with lower slope and with features similar to case 1^44,45^. The Weibull’s model in equation 2 can be linearized as;

3 $$\mathrm{log}\,[-\,\mathrm{ln}(1-{\rm{M}})=b\,\mathrm{log}\,(t-{T}_{i})-\,\mathrm{log}\,a\,]$$

The plot of log–log of - In (1-M) versus time (t) gave a straight line with shape parameter (b) obtained from the slope, and the diffusion/release process time parameter (*a*) obtained from the intercept corresponding to the ordinate (1/*a*) at time t = 1. The fitting results show that the release of the inhibitor compounds loaded in HNTs followed the case 3 as b< 1 (Table SI2). This agrees well with the nature of the various release profiles considering the recorded initial fast release of the inhibiting species loaded in the outer pores/lumens of HNTs followed by the slow release of compounds loaded inside the lumen. The release process time (*a*) increased remarkably from 2–3 h for the naked inhibitors crystals dissolution in water to 10–20 h for the release of inhibitors loaded in HNTs and up to 200 h for the complete release of inhibitors loaded in HNTs capped with Fe^3+^ and CTS tubular encapsulation. The remarkable decrease in the diffusion coefficients (K), and the corresponding increase in the release process time (*a*) (especi*a*lly for loaded HNTs with interfacial treatments) would be beneficial in real field applications where sustainable active feed-back functionality is required.

### Coating preparation, morphology, and physical properties

The innovative active feedback and self-repairing epoxy coatings were prepared by dispersing 5–10% active agents loaded HNTs into epoxy coating as described in the method section. Although HNTs is highly hydrophilic^46,47^, its dispersal in epoxy coating did not alter the wettability of the coating. The average contact angles and the associated water droplet images for epoxy and epoxy coatings embedded with 5–10% of HNTs are presented in Fig. SI5. The results indicate that addition of HNTs slightly increased the average water contact angles for the composite coatings. The increase in the water contact angles could be related to the increase in coating’s surface roughness due to the embedded nanoparticles^48^. This is supported by the AFM images and the associated average roughness parameters depicted in Fig. SI6 of the supporting material, which shows that the average surface roughness of epoxy coating increased slightly with the addition of different amounts HNTs. The SEM images in Fig. 3c(i–iii) compares the micromorphology for epoxy coating (i), epoxy coating embedded with 10% loaded HNTs nanotubes (ii), and epoxy coating embedded with 5% loaded HNTs nanotubes (iii). Uniformly dispersed and well-embedded nanoparticles can be observed (Fig. 3cii–iii). Such a well distribution of HNTs nanotubes in the epoxy matrix assures the effective release of the loaded active agents upon coating breach or damage (defects). Therefore, the various electrochemical and spectroscopic experiments were carried out on the following: (a) epoxy reference coating (Ep), (b) epoxy coating embedded with 5% HNTs loaded with BTA (Ep–HNTs–BTA), (c) epoxy coating embedded with 10% HNTs loaded with MBT (Ep–HNTs–MBT), (d) epoxy coating embedded with 5% HNTs loaded with BTA with Fe stopper (Ep–HNTs–BTA–Fe), (e) epoxy coating embedded with 10% HNTs loaded with MBT with Fe stopper (Ep–HNTs–MBT–Fe), (f) epoxy coating embedded with 5% HNTs loaded with BTA with CTS encapsulation (Ep–HNTs–BTA–CTS), and (g) epoxy coating embedded with 10% HNTs loaded with MBT with CTS encapsulation (Ep–HNTs–MBT–CTS).

### Electrochemical impedance spectroscopy (EIS data)

**Figure 4:**
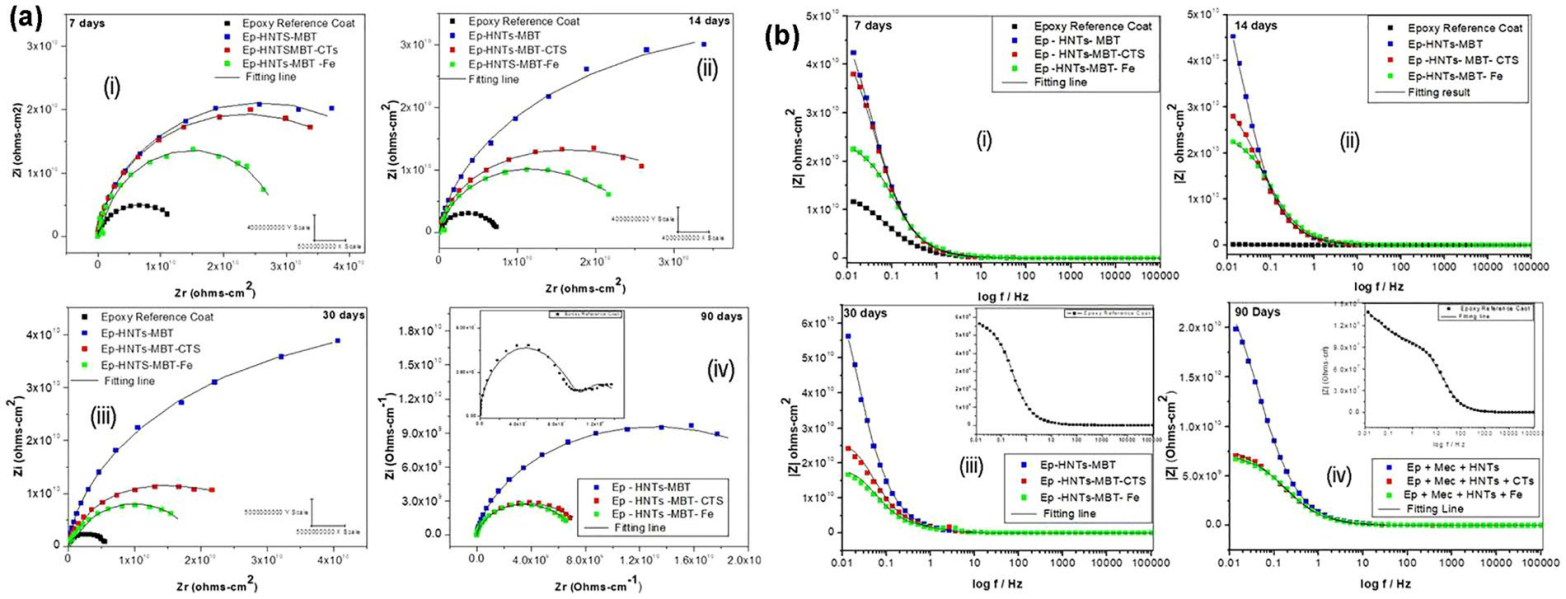
(**a**) Nyquist impedance spectra of coating/metal systems for; Ep, Ep–HNTs–MBT, Ep–HNTs–MBT–CTS and Ep–HNTs–MBT–Fe, after different immersion times: (i) 7 days (ii) 14 days (iii) 30 days (iv) 90 days. (**b**) Bode impedance modulus plots of coating/metal systems for; Ep, Ep–HNTs–MBT, Ep–HNTs–MBT–CTS and Ep–HNTs–MBT–Fe after different immersion times: (i) 7 days (ii) 14 days (iii) 30 days (iv) 90 days.

The long-term passive barrier properties of epoxy reference coating (Ep) and the modified epoxy coatings applied on Q235 carbon steel exposed in 3.5% NaCl were evaluated with EIS technique. The various Nyquist plots for the impedance responses of the carbon steel samples coated with: Ep reference coat, Ep doped with HNTs–MBT, Ep doped with HNTs-MBT-CTS and Ep doped with HNTs-MBT-Fe, after different immersion times are presented in Fig. 4a(i–iv), while the corresponding Bode modulus plots are presented in Fig. 4b(i–iv).

**Figure 5:**
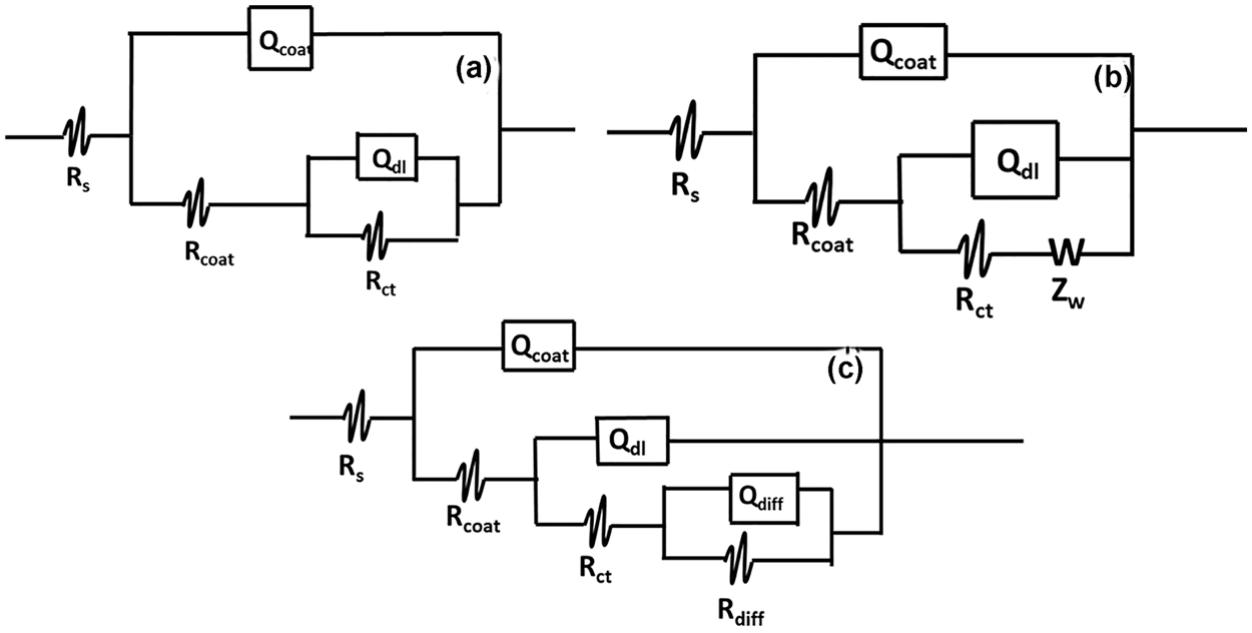
The equivalent electrical circuits used for describing the impedance response of epoxy and the various modified epoxy composite coatings: (**a**) with two time constants (**b**) with two time constants plus Warburg impedance (**c**) with three time constants.

**Figure 6:**
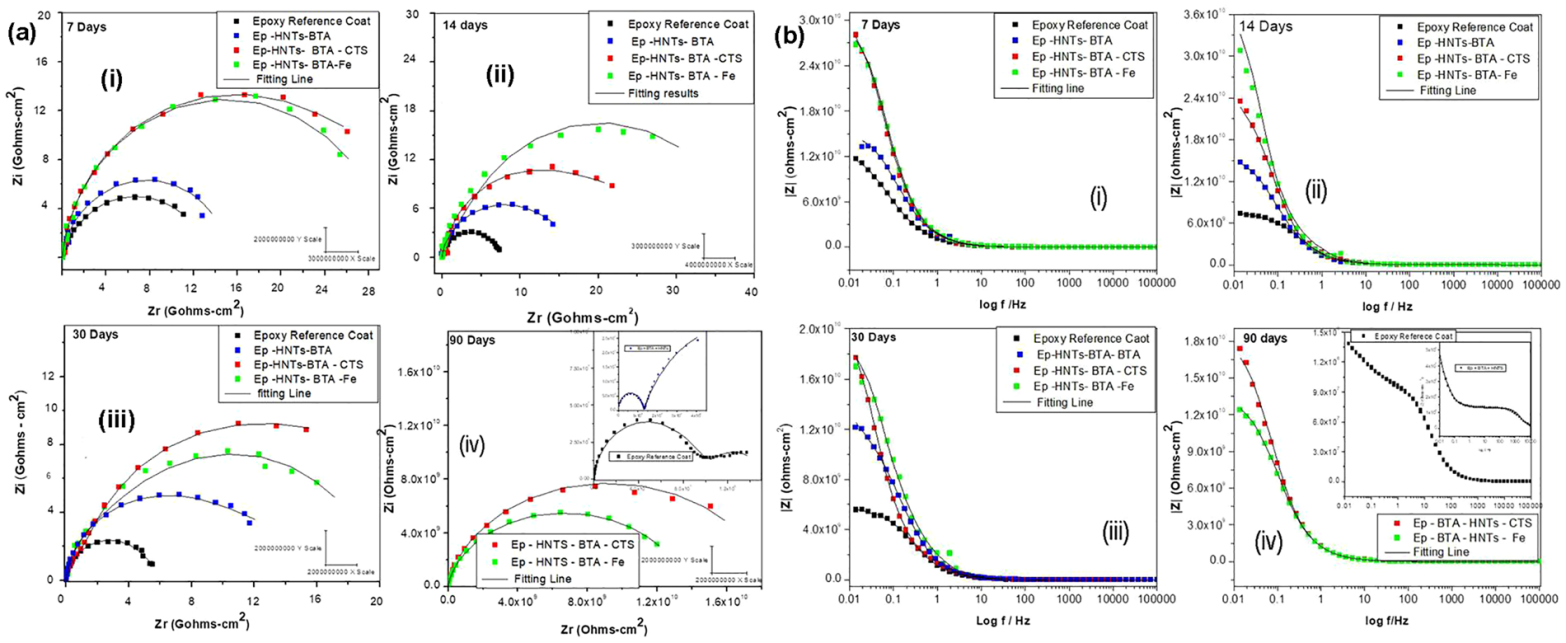
(**a)** Nyquist impedance spectra of coating/metal systems for; Ep, Ep–HNTs–BTA, Ep–HNTs–BTA–CTs and Ep–HNTs–BTA–Fe for after different immersion times: (i) 7days (ii) 14 days (iii) 30 days (iv) 90 days. (**b**) Bode impedance plots of coating/metal systems for; Ep, Ep–HNTs–BTA, Ep–HNTs–BTA–CTs and Ep–HNTs–BTA–Fe after different immersion times: (i) 7days (ii) 14 days (iii) 30 days (iv) 90 days.

Three equivalent circuits depicted in Fig. 5(a–c) were employed to appropriately analyze the various impedance data. The electrical circuit in Fig. 5a comprises of the electrolyte resistance (R_s_), constant phase elements representing the coating capacitance (CPE_c_), the coating resistance to the passage of electrolytes (R_po_), the constant phase elements representing the double layer capacitance between the metal surface/electrolyte solution (CPE_dl_) and the charge transfer resistance across the metal surface (R_ct_). The equivalent circuit presented in Fig. 5b has an additional diffusion component (the Warburg finite diffusion impedance element), while the equivalent circuit in Fig. 5c exhibited three-time constants – the third constant composed of diffusion capacitive component (CPE_dff_) and resistance (R_diff_). Meanwhile, the Warburg impedance (Z_W_) in the second circuit (Fig. 5b) represents the diffusion of corrosive ions within the coating pores^49,50^. The electrical circuit in Fig. 5a was adopted to mostly fit the impedance data for the first seven days of immersion, the circuit in Fig. 5b fitted most of the impedance after seven days; while the circuit in Fig. 5c fitted only epoxy reference coating after 60–90 days of immersion. The various equivalent circuits contain 2 and 3 time constants indicating that water molecules have reached the metal/coating interface even before seven days of immersion and have initiated somewhat electrochemical reaction^37,50^. However, the reason that only single capacitive loop is phenomenally visible in most of the Nyquist and Bode impedance plots (except for epoxy after 90 days) is likely that the initiated electrochemical reaction area at the metal/coating interface is probably not significantly pronounced at these systems; causing the overlap of the time relaxation of coating impedance and impedance of the electrochemical reaction at the metal/coating interface^51^. However, after longer immersion period (above 30 days), the impedance spectra for epoxy reference coating changed obviously into two conspicuous time constants which can be ascribed to significant accumulation of corrosion products at the metal/coating interface, and the circuits in Fig. 5a,b could no longer fit the plots. Thus, another equivalent circuit Fig. 5c was adopted to accommodate the diffusion impedance of the accumulated corrosion product species. In this case, the diffusion process of the corrosion products across the coating barrier may become a control factor in the Faradaic process – which is usually not ideal Warburg impedance. And this is responsible for the recorded deviation of the dispersive number, n, from 0.5 ^51^. Also, it is also important to note that, in the various equivalent circuits, the constant phase element (CPE) has been used to replace the capacitance elements to accommodate the scattering effect due to surface inhomogeneity of the coating surface. As visible from the fitting lines of the various impedance spectra in Figs 4 and 6(i–iv), good fittings were obtained with Chi-square (Χ^2^) ranging from 3 × 10^−3^ − 4 × 10^−4^.

It is well documented previously in the literature that the magnitude of impedance modulus at low frequency (|Z|_0.01_ Hz) is an appropriate parameter for evaluating the overall corrosion protection performance in organic protective coatings^52,53^, while the charge transfer resistance (*R*_*ct*_) reflects the resistance to electron transfer reaction at the metal surface underneath the coating – which is inversely proportional to the undercoating corrosion rate^12,49–53^.

Thus, the performance of our various coating formulations over different immersion times can be evaluated from the magnitude of impedance modulus at low frequency |Z|_0.01_ Hz (which can be read from the various Bode modulus plots) and the values of the charge transfer resistance (*R*_*ct*_) (which can be read from the abscissa of the Nyquist impedance plots – the diameter). These two predictive parameters were preferentially chosen to interpret the impedance results because of the complex nature of our overall impedance data. Close scrutiny of the various impedance spectra in Fig. 4a,b revealed two striking features; (a) addition of MBT-HNTS, MBT-HNTs-CTS and MBT-HNTs-Fe into the epoxy coating appreciably increased the diameter of the Nyquist plots and the magnitude of the low frequency impedance modulus (|Z|_0.01_ Hz) against the reference epoxy coating following the order: MBT-HNTs > MBT-HNTs-CTS > MBT-HNTs-Fe > Ep., (b) The magnitude of |Z|_0.01_ Hz decreased steadily in the Ep reference coating down to |Z|_0.01_ Hz of 10^8^ Ω cm^2^ after prolonged exposure time (90 days), whereas, in the various modified epoxy coatings, the magnitude decreased insignificantly, thus maintaining (|Z|_0.01_ Hz) around 10^10^ Ω cm^2^ throughout the studied experimental times. The drop of |Z|_0.01_ Hz of Ep reference coating from 10^10^ Ω cm^2^ to 10^9^ Ω cm^2^ after 30 days and down to 10^8^ Ω cm^2^ after 90 days indicates that the corrosive ions of the electrolyte solution at the metal/coating interface have initiated reasonable corrosion action. However, the recorded high value of low frequency impedance magnitudes (|Z|_0.01_ Hz = 10^10^ Ω cm^2^) by the modified epoxy coatings (MBT-HNTS > MBT-HNTs-CTS > MBT-HNTS-Fe) even after 90 days of immersion can be attributed to the combined effects of improved coating compactness/barrier properties by the dispersed HNTs (slow permeation of electrolyte solution) and the slow release of the MBT inhibitor loaded in the HNTs as water percolates the coating micro-pores.

Similar observations were recorded with Ep reference coating modified with BTA loaded HNTs presented in Fig. 6a(i–iv) for Nyquist plots and Fig. 6b(i–iv) for Bode modulus plots. For instance, the |Z|_0.01_ Hz of 2.8 × 10^10^ Ω cm^2^, 2.7 × 10^10^ Ω cm^2^ and 1.3 × 10^10^ Ω cm^2^ recorded for BTA-HNTs-CTS, BTA-HNTs-Fe, and BTA-HNTs respectively after 7 days of immersion in 3.5% NaCl solution only showed infinitesimal decrease to 1.2 × 10^10^, 1.8 × 10^10^ Ω cm^2^ and 1.7 × 10^10^ Ω cm^2^ respectively after 30 days of immersion, whereas, the reference Ep coating had decreased to 10^9^ Ω cm^2^.

However, when the immersion period was extended to 90 days, the low frequency impedance modulus (|Z|_0.01_ Hz) for BTA-HNTs decreased to 4.7 × 10^6^ Ω cm^2^ lower than the |Z|_0.01_ Hz value for the epoxy reference coating (worse performance) at 10^8^ Ω cm^2^. This sharp drop in |Z|_0.01_ Hz for BTA-HNTs after prolonged exposure period is likely caused by the excessive leaching of BTA which is very soluble in water. According to Zheludkeick *et al*.^54^, the leaching of high soluble compounds in coatings can create an osmotic pressure leading to blistering and delamination of the protective coatings. The osmotic pressure can, therefore, increase the rate of water ingress/accumulation within and beneath the coating, thereby destroying the barrier integrity. Interestingly however, Ep with BTA loaded HNTs interfacially treated with CTS encapsulation and Fe^3+^ tube end stopper (BTA-HNTs-CTS and BTA-HNTS-Fe) maintained the high value of low frequency impedance modulus of |Z|_0.01_ Hz within 10^10^ Ω cm^2^ even after the 90 days of immersion. This clearly confirms that CTS encapsulation and Fe^3+^ tube end capping effectively mitigated the leaching and release rates of water soluble BTA from the loaded HNTs into the surrounding electrolyte solution and within coating matrix over prolonged exposure times. This observation agrees well with the release profile plots in Fig. 2a and the various data from the theoretical model predictions in Table SI 2 of the supporting material in affirming the importance of incorporating control and/or smart release systems in active anti-corrosion protective coatings.

**Figure 7:**
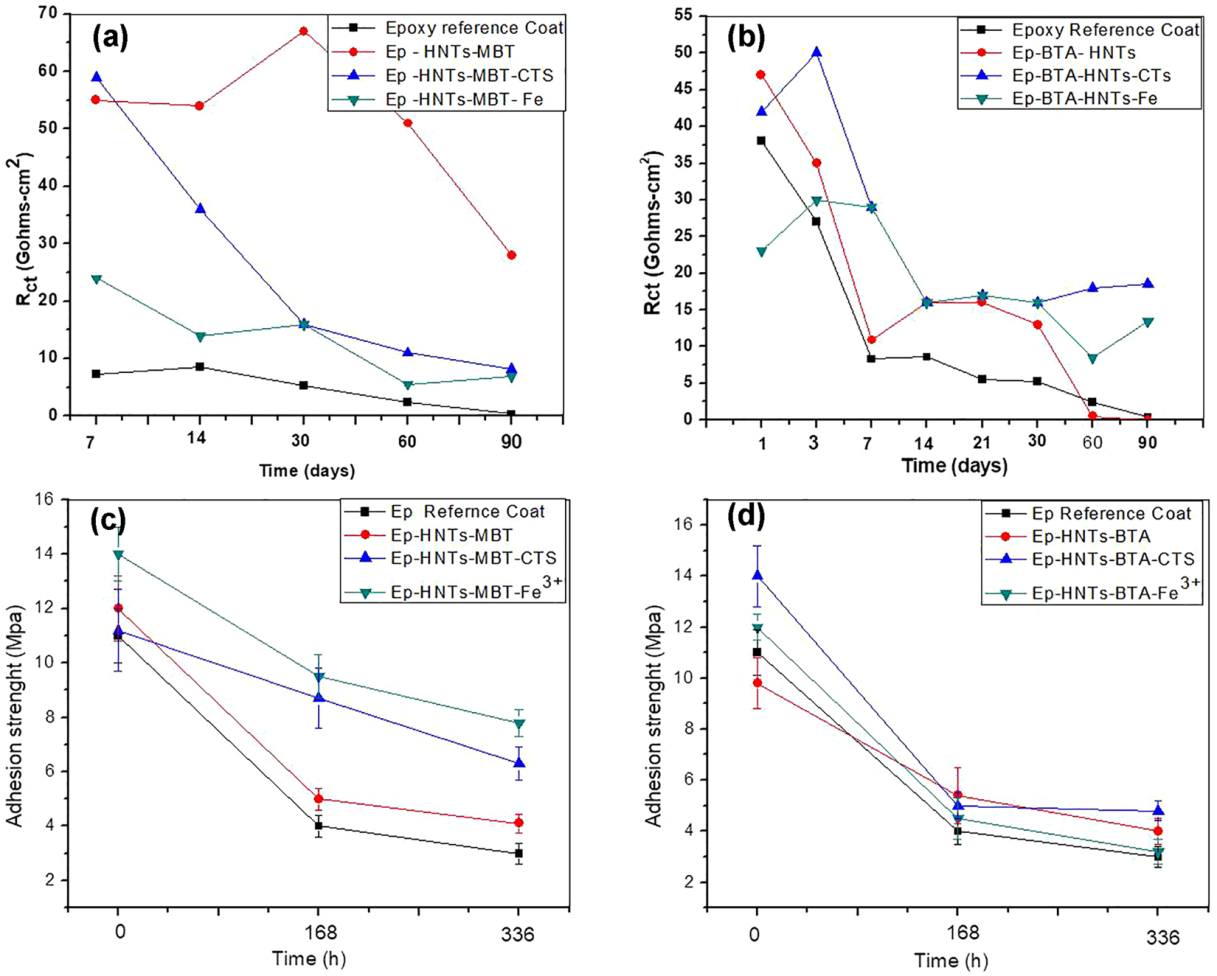
Comparison of the evolution of charge transfer resistance (*Rc*_*t*_) over different immersion time for epoxy reference coating (Ep) and the various modified coatings containing MBT loaded HNTs (**a**), epoxy reference coating with the various modified coatings containing BTA loaded HNTs (**b**), the wet adhesion strengths over different immersion times for epoxy reference coating and the various modified coatings containing MBT loaded HNTs (**c**), and epoxy reference coating with the various modified coatings containing BTA loaded HNTs (**d**).

Going further to clarify the protective performances of the various coatings, the charge transfer resistance was plotted over time in Fig. 7a,b. The results show that epoxy coatings doped with MBT-HNTs, MBT-HNTs-CTS and MBT-HNTs-Fe (Fig. 7a) and epoxy coatings doped with BTA-HNTs, BTA-HNTs-CTS and BTA-HNTs-Fe (Fig. 7b) had higher *R*_*ct*_ values compared to the Ep reference coating. The higher *R*_*ct*_ values recorded with the various modified coatings reflect mitigation of the corrosion process at the metal/coating interface by the released active species. Thus, the various impedance data collectively show that the well-dispersed active species loaded HNTs (including the HNTs samples with interfacial modifications), appreciably improved the barrier and anti-corrosive protective performance of epoxy the coating.

### The undercoating corrosion and adhesion data

**Figure 8:**
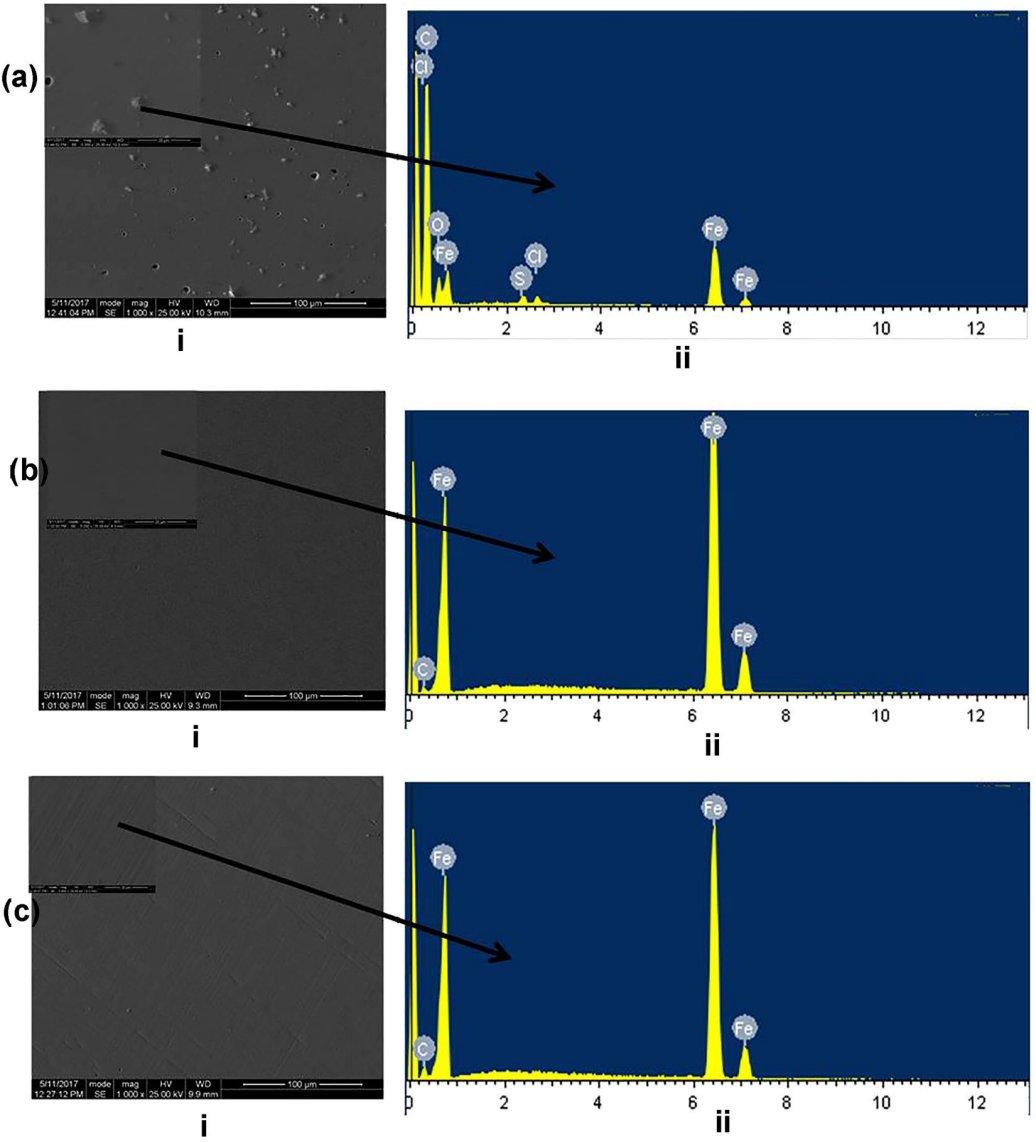
SEM/EDS data of the steel surfaces after peeling the applied coatings for the coated samples immersed in 3.5% NaCl after 14 days; (**a**) epoxy (**b**) epoxy doped with MBT loaded HNTs and (**c**) epoxy doped with BTA loaded HNTs.

To evaluate the extent of corrosion propagation underneath the coatings and the effect on adhesion, SEM and pull-off wet adhesion tests were conducted on the steel panels coated with Ep reference coating and the various modified epoxy coatings. Figure 8 compares the SEM images of the surface morphologies of steel samples coated with (a) Ep reference coating, (b) Ep doped with MBT-HNTs and (c) epoxy doped with BTA-HNTs (C), after 14 days of immersion in 3.5% NaCl solution (The images were taken after the pull-off of the applied surface coatings was carried out using pull-off adhesion testing machine). Also, the photographs comparing the physical appearance of the steel surfaces (after the pull-off of the coatings by the adhesion tester) are shown in the supporting information (Fig. SI7). The SEM images (Fig. 8) and the photographs (Fig. SI7), collectively reveal lots of corrosion pits and products for the panel coated with the epoxy reference coating, whereas, the panels coated with the modified epoxy coatings did not show noticeable corrosion pits and products indicating an active corrosion protection. The EDS data for the panel coated with Ep reference coating in Fig. 8, indicate that the corrosion products composed of oxides and chlorides of iron as expected considering the nature of the studied electrolyte solution.

The plots in Fig. 7c,d compares the average adhesion strengths (MPa) over different immersion times for Ep reference coating and the various modified active epoxy coatings. Inspection of the plots clearly revealed that the introduction of both the HNTs loaded with BTA and MBT active agents and the loaded HNTs with interfacial treatments with Fe^3+^ and CTS increased the adhesion strengths when compared with the Ep reference coating. Adhesion of coatings on substrates under immersion (wet adhesion) condition depends among other things on the rate of water ingress/accumulation in the coating matrix and the degree of formation/accumulation of corrosion products at the metal/coating interface. Thus, the higher average adhesion values over different immersion times for the modified coatings can be related to enhanced compactness (electrolyte permeation resistance) and mitigation of undercoat corrosion. This observation agrees well with the impedance and microscopic data in revealing the combined passive and active anti-corrosive performances of the various innovative epoxy coatings.

### Active feedback and self-healing assessments

**Figure 9:**
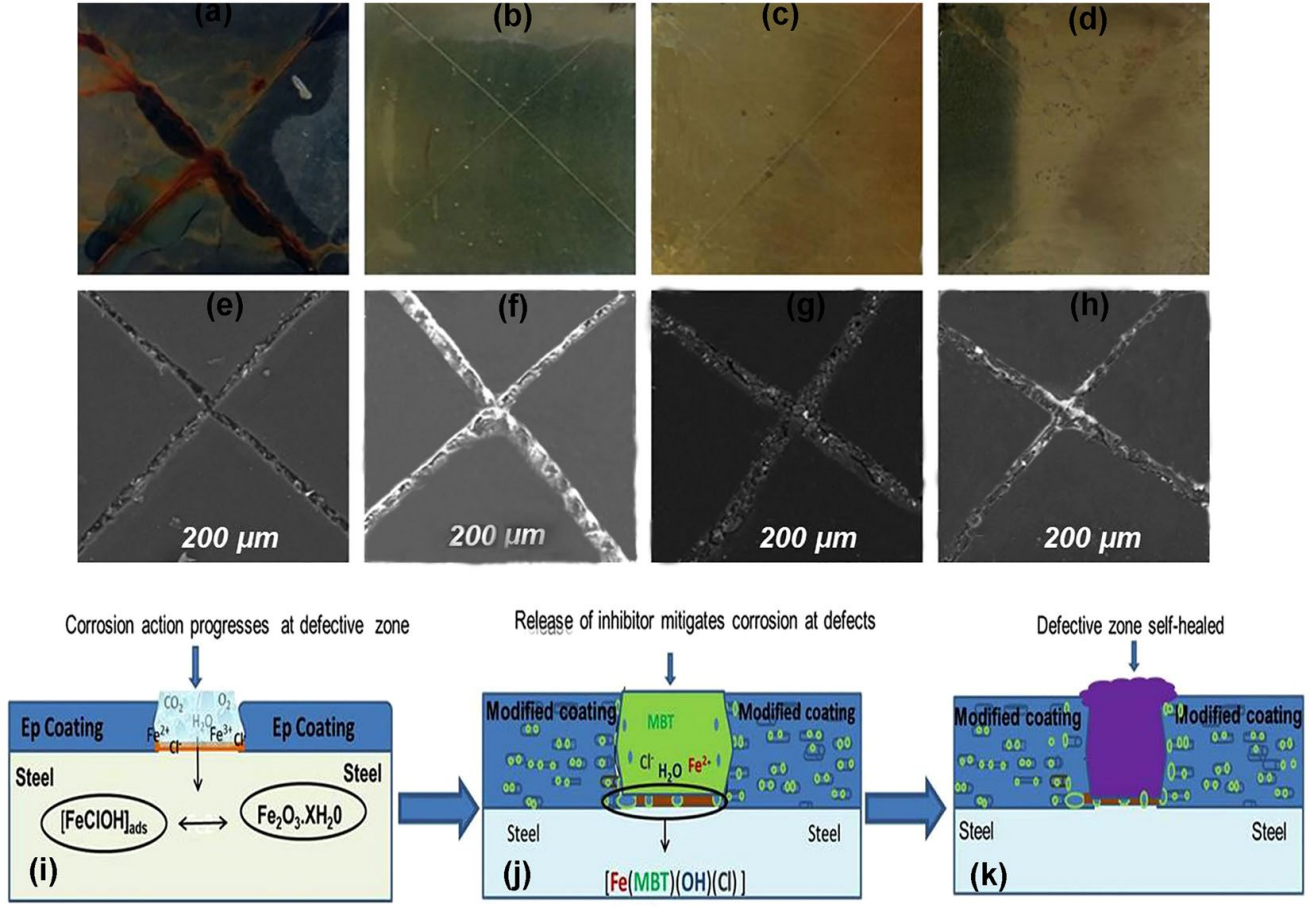
(**a**–**d**) Photographs comparing the active feedback anticorrosion performances of different epoxy composite coatings applied on Q235 steel panels after 336 h exposure in a salt spray chamber: (**a**) epoxy reference coating (**b**) epoxy doped with MBT loaded HNTs (**c**) epoxy doped with MBT loaded HNTs encapsulated with CTS and (**e**) epoxy with MBT loaded HNTs capped with Fe, and the corresponding SEM images (**e**–**h**) revealing the healing abilities after 168 h immersion in salt solution, and also the scheme illustrating the process of corrosion in reference epoxy (**i**), the modified epoxy coatings (**j**) and the self-healing of defects (**k**).

Close scrutiny of the photographs and SEM images in Fig. 9(a–h), and Fig. SI8(ai–av)–(bi–bv), reveals a combination of active mitigation of corrosion reactions (prevention of iron rust formation) around the artificially scribed areas and the closing of the scribed areas (enhanced self-healing) for the panels coated with the modified epoxy coatings compared with the panels coated with Ep reference coating. The active feedback functionality is duly attributed to the slow release of the inhibiting compounds loaded in the HNTs dispersedly embedded in the epoxy coating. The released inhibitive species saturates the crevices of the scribed areas and forms a stable protective inhibitor complex film that isolates the metal surface from the corrosive environment (unlike in the reference epoxy coating where corrosion reaction progresses without hindrance) as shown in the scheme in Fig. 9(i–k). On the other hand, the observed enhanced self-healing effects exhibited by the modified coatings can be related to; (a) the reduction of the delamination action at the defective zones by the free-moving active agents released by the coating system, and (b) the promotion of the epoxy self-healing tendency by CTS polymer gels.

#### Mechanisms of the active feedback and self-healing effects

The mechanisms for the active corrosion mitigation recorded with the various modified epoxy coatings have been illustrated in the scheme in Fig. SI9, and can be summarized as follows:

- the released active compounds adsorb on the surface of the exposed steel surface by replacing the water molecules previously occupying the steel surface as shown^55,56^;
  4a $${{\rm{Inh}}}_{({\rm{sol}})}+{{\rm{xH}}}_{2}{{\rm{O}}}_{({\rm{ads}})}\to {{\rm{Inh}}}_{({\rm{ads}})}+{{\rm{xH}}}_{2}{0}_{({\rm{sol}})}$$
  (Where x represents the number of water molecules replaced by one molecule of adsorbed inhibitor species).
- the adsorption of the released active compounds at the sites previously attached by Cl^−^ on the steel surface could result in the formation of a more stable and protective Fe-chloro-inhibitor complex film which can hinder the migration of corrosive species to the bare metallic surface as represented by equation 4b ^55^;
  4b $${{\rm{Fe}}}^{2+}+{{\rm{xH}}}_{2}{\rm{O}}+{{\rm{yCl}}}^{-}+{\rm{z}}({\rm{Inb}})\leftrightarrow {[{\rm{Fe}}{({\rm{Inh}})}_{{\rm{z}}}{({\rm{OH}})}_{{\rm{x}}}{({\rm{Cl}})}_{{\rm{y}}}]}^{2-x-y}+{({\rm{H}})}^{+}$$
- the protonation of some inhibitor species by the proton released at the anodic regions during the onset of corrosion reactions could initiate electrostatic interaction between the protonated inhibitor compounds and the negatively charged metal surface. The negative charge of the steel surface is related to the electron loss associated with the anodic sites and/or the pre-adsorption of Cl^−^ on the steel surface. Thus, the driving force for the reactions represented in equations 4a–b is directly related to the donor-acceptor interactions between electron rich regions in the neutral inhibitor molecules (such as the л electrons, N and S atoms) and the vacant d-orbital of iron; whereas, the driving force for the electrostatic adsorptive interaction is related to the local acidification reaction associated with the onset of corrosion reactions at the anodic areas. These proposals indicate that the local acidification (pH reduction) during the onset of corrosion reactions at the defects and at steel/coatings interface could increase the corrosion mitigating efficacies of organic active compounds even in neutral solutions. This is probably responsible for the almost non-appearance of rust and pits observed with the modified coatings despite the nature of the electrolyte (neutral salt solution).

The mechanisms for the recorded enhanced self-healing effects exhibited by the modified coatings compared with the reference epoxy coating (Fig. 9e–h and Fig. SI8b i–v) can be summarized as follows: (a) although epoxy has an inherent self-healing ability (compare the fresh cut images in Fig. SI8ai and bi with the corresponding images after 24 and 168 immersion times in Fig. SI8 and Fig. 9), the presence of BTA and MBT inhibitors including the HNTs inhibitor loaded samples capped with Fe^3+^seems to enhance this effect, which can be attributed to the effect of the released inhibitors on the delamination rate at the scribed areas and (b) the obvious self-healing effect observed in the presence of CTS has been previously attributed to the protonation of the amine group of chitosan in aqueous medium at lower pH (pH < 6)^57^^,^^58^, which causes swelling due to chain dissociation and charge repulsions. Then, the secondary interactions which proceed these effects such as molecular entanglements, hydrogen bridges, surface rearrangements and randomization between the acetylated units of chitosan can lead to the formation of more solid-like hydrogel in a process formerly referred to as polymer-polymer interface reaction^24,59^.

### Physical performance assessments

Although, the dispersal of clay HNTs clay nanoparticles in polymer coatings has been reported to improve the physical/mechanical properties of the coatings^10,26,27^, dry adhesion and impact test were carried out to afford further insights on the physical properties of the coatings. The crosscut dry adhesion test was conducted according to the ASTM D 3002 and ASTM D 3359 standards (to compare the dry adhesion properties of the various coating formulations on the steel surface), while impact test (SAC–GB/T 1732–93 standard) was carried out to investigate ability of the various coatings to resist cracking, shattering (due to brittleness), or chipping upon rapid deformation. The various photographs comparing the images of the cross–cut of the various coatings with the standard presented in Fig. SI10 revealed that the edges of the cut are smooth and none of the squares of the lattice was detached, indicating that the cross cut dry adhesion data for both the Ep reference coating and the various modified coatings are similar and followed the same class (ISO Class 0 / ASTM –Class: 5B). The results of the impact tests (photographs presented in Fig. SI11 of the supporting material) reveal that there were no shattering as a result of coating brittleness and the various coatings peeled uniformly and similarly. This indicates that the positive physical properties of the coatings were not sacrificed by the introduction active feedback / self-repairing systems.

## Conclusions

*In-situ* EIS, SEM and optical techniques have been adopted to successfully probed the possible long-term combined active feedback, self-healing and passive protective performances of a new class of innovative epoxy coatings, including their failure behaviors over lengthened exposure time. The EIS data revealed the improved passive barrier protective functionality of the innovative coatings while the active feedback and self-healing functionalities were confirmed by the following: (a) the non-appearance of iron rust at the artificially scribed areas of the coated panels exposed in salt spray chamber, (b) the absence of rust and pits on the steel/coating interface of panels immersed in salt solution over lengthened exposure time, and (c) the enhanced self-healing of the artificially scribed areas of the panels exposed in both the salt spray chamber and salt solution. BTA and MBT active species were successfully loaded into the clay nanotube and high loading efficiency (up to 8% for MBT and 12% BTA inhibiting agents) which assures long-lasting active corrosion protection action was recorded by TGA / DTG measurements. Fe^3+^ and chitosan-glutaraldehyde polymer films respectively proved to be excellent alternative tube end stopper and polymer encapsulating agent for impacting sustainable control release of active species loaded in HNTs nanotubes. Interestingly, the observations made from the EIS results confirmed the ability of the Fe^3+^ tube end stopper and CTS polymer encapsulation to mitigate the leaching rate of high soluble BTA over lengthened immersion time following the results of the release profiles and the theoretical predictions. The overall experimental data indicate that the fabricated new class of epoxy coatings excellently exhibited combined passive, active and self-healing protective actions, and according to the various physical / mechanical tests, did not undermine the inherent physical properties of Ep coating –a feature that increases the potential industrial application.

## Materials and Methods

### Materials

Halloysite clay nanotubes (HNTs) were purchased from Natural Nano (Inc. Pittafor, New York 14534) and were used without further treatments (as-received). Benzotriazole (BTA) and 2-mercaptobenzothiazole (MBT) used as active species were supplied by the Aladdin Industrial Cooperation, while sodium chloride, FeCl_3_.6H20 (hydrated iron (III) chloride), chitosan, 25% glutaraldehyde solution, and xylene were supplied by Sinopharm Chemical Reagents Co. Ltd. The reference epoxy coating; E44 epoxy resin (binder), and polyamide (curing agent) were supplied by TY-650, Tianjin Yanhai Synopharm Chemicals. The steel sample used as the substrate was Q235 carbon steel with nominal composition: C–0.14–0.22, Mn–0.30–0.65, Si–0.30, S–0.08, P–0.045 and Fe–margin. The steel samples for the electrochemical tests were embedded into epoxy resin (after welding with copper wire as an electrical connector) and allowed to cure; exposing only one surface with area of 1 cm^2^. The metal coupons were all polished with emery papers from 150 up to 600 grit, degreased in ethanol and dried in warm air. The electrolyte solution employed for the various test was 3.5% NaCl.

### Characterization of the as-received halloysite nanotube sample

Characterization of the halloysite clay nanotubes was carried out using infra-red spectroscopy (FTIR, Magna–IR 560 spectrometer) and X-ray diffraction spectroscopy (Rigaku–D/max 200 diffractometer with a Cu K_alpha_ target). The HNTs surface morphology was evaluated with scanning electron microscopy (SEM model XL30FEG) after initial coating with Cu (~1 nm) using a sputter coater (BALTEC-GED 030 carbon evaporator), while the volume and lumen sizes were determined with transmission electrode microscope (TEM, JEM-2100F, JOEL, Tokyo, Japan) operated at 120 KV. The particle size distribution and surface charge for over different pH ranges were recorded with Malvern ZEN3600 Zetasizer. The UV–visible spectrophotometer (Ray Leigh VV–2100) was used to obtain the absorption spectra and concentrations of the inhibitors. The maximum absorbance for BTA and MBT was found to be 258 nm and 320 nm respectively. Thus, calibrations and subsequent concentration determinations were analysed at these points.

### Tube loading

The loading of the as-received HNTs samples was carried out under vacuum (0.1 MPa) using a vacuum pump (SHZ–D(111), Yuhua company) as demonstrated in the Fig. SI12 presented in the supporting information. HNTS powder (~10 g) were sieved with a 120 μm mesh to remove possible aggregates and soaked in a 500 ml saturated inhibitor solution in acetone in a 1000 ml vacuum flask. The suspension was sonicated for 1 hour and thereafter allowed to stand for another 2 hours to intensify the soaking of inhibitor solution with the nanoparticles before applying vacuum. During loading, the solution was placed under vacuum for 3 h and broken for 2 h to allow the expulsion of air bubbles trapped within the HNTs powder and ingress of concentrated inhibitor solution into the lumen and pores (slight fizzing of the suspension under vacuum indicates the removal of air with subsequent replacement with concentrated inhibitor solution). The vacuum creation and breaking processes were repeated 4–5 times until the inhibitor/HNTs suspension was evaporated into a slurry mixture. Then, the slurry was allowed to stand for 24 h so that the loaded inhibitor could crystallize in the pores and lumen of the HNTs. Finally, the HNTs were separated by washing quickly with acetone (to collect the un-loaded inhibitors compounds) and centrifuged for 2 minutes. The separated loaded HNTs particles were further re-washed with water (to minimize the amount of exogenously adsorbed inhibitor compounds) and separated like before and dried in an electrical oven at 40 °C.

### Kinetics of corrosion inhibitor release

The release experiments were carried out on ~0.5 g of inhibitor loaded HNTs in 250 ml de-ionized water (DI) of pH 6.5–7.5 at room temperature. To establish a well dispersed suspension of the loaded HNTs in the aqueous solution the suspension was constantly stirred slightly with a magnetic stirrer during the release process. The concentration of the released inhibitor over time was monitored with UV-visible spectrophotometer. The sample for analysis (1 ml) was syringed from the suspension at the end of every 1 h interval, diluted and filtered through micro-pore filter paper to prevent the passage of light scattering aggregates before UV-vis measurements. To maintain constant volume, equivalent 1 ml of water was added to replace the amount taken for the UV experiment. At the end of the release experiment, the complete release (assumed to be 100%) was obtained by further diluting the suspension 4 times its initial volume with water and sonicating for 12 h until no noticeable increase in absorbance due to inhibitor release was observed. To obtain the diffusion profile for the naked inhibitors in water (for comparison with the release profile from loaded nanotubes), the bulk inhibitor crystals was dissolved in water and the concentration of the diffused species was monitored with UV-visible spectrophotometer until a saturation level is reached.

### Controlled release experiments

Tube-end capping with Fe^3+^ was achieved by dispersing loaded and dried samples of HNTs in 0.08 M FeCl_3_.6H_2_O solution for 1 min followed by instant separated by centrifugation, dried, ground and sieved as before. CTS Polymer film encapsulation was achieved by exposing the inhibitor loaded HNTs dried powder in a chitosan solution buffered appropriately for 10–15 minutes. Then, 10–20 ml of 10% glutaraldehyde solution was added drop-wise to cross-link the electrostatically adsorbed chitosan polymer on the surface of HNTs.

### Coating preparation, application, and physical tests

The reference epoxy coating used in this study is consistent with a stoichiometric mass ratio of 1: 0.8: 0.6 for the binder, curing agent and solvent respectively. The composite epoxy coatings with embedded active species loaded HNTs were prepared by dispersing the loaded HNTs in the binder–solvent solution using a magnetic stirrer to ensure complete dispersal and separation of aggregates before the addition of the curing agents. The composite mixture was allowed to stand for up to 0.5 h before application on the steel surface. The paint mixture was applied on the steel samples using a hand brush. Two layers were applied (each layer approximating 110 μm thickness) at the interval of 1 h, to ensure the closure of some micro-pores by air bubbles. Thus, the average coating thickness of about 220 ± 10 μm was obtained. The coating thickness was measured with a portable electronic coating gauge (Positector 6000, Defelsko) according to ISO 2808 standard procedure. The applied coatings were allowed to dry in the oven with the following conditions: 40 °C for 12 h, 60 °C for 48 h and room temperature for up to 4 days. The coatings micro-morphologies were evaluated with SEM and atomic force microscopy (AFM–Picoplus 2500 with scanning probe microscopy). The wettability of the coating surface was measured with JCY contact angle meter equipment model 306207. The wet adhesion of the coating applied on the steel surface was measured with Positest pull-off adhesion tester (ASTM D4541–02). The resistance to cracking and shattering was evaluated according to SAC-GB/T 1732-93, while the cross–cut dry adhesion testing was analyzed according to ASTM D 3359.

### The electrochemical measurements

EIS experiments were performed by means of AUTOLAB electrochemical measurements system using a three-electrode cell. A platinum foil of dimension 2 cm × 2 cm and a saturated calomel electrode (SCE) served as a counter electrode and reference electrode respectively. The steel samples that were covered with epoxy resin leaving only 1 cm^2^ surface area exposed were coated with the appropriate coating formulation and served as the working electrode. All EIS experiments were recorded after an initial automatic 0.5 h open circuit potential (OCP) recording intended to ensure the stability of the systems. The frequency range was from 100 kHz to 10 mHz with signal amplitude perturbation of 30 mV. The various impedance data were analyzed using ZSimpWin 3.10 software developed by the Priceton Applied Research.

### Salt spray chamber tests

The active feedback anti-corrosive property of the reference epoxy and the various modified epoxy coatings were evaluated through pictorial examination of the iron rusts formation on the artificially scribed areas of coated steel panels exposed in a salt spray/prohesion/humidity chamber performed according to ASTM D1654 standard salt spray test method. The salt solution used was sodium chloride (5 wt. %). The coated panels were carefully and slightly scratched (uniformly) with a blade of thickness (0.40 mm ± 2) down to the metal surface (without destroying the surface terrain) and placed at an angle of 30^0^ in the chamber. After 168–336 h exposure times, the surface of the panels was photographed with a digital camera and presented. Self-healing assessment test was conducted using SEM on coated samples (slightly cut) immersed in 3.5% NaCl after 0 h, 24 h, and 168 h.

### Data availability

All data generated or analysed during this study are included in this published article (and its Supplementary Information files).

## Electronic supplementary material

Supporting Information
