# Supplementary material for: Understanding the anticorrosive protective mechanisms of modified epoxy coatings with improved barrier, active and self-healing functionalities: EIS and spectroscopic techniques
Source: Sci Rep. 2017 Nov 15;7:15597. doi: 10.1038/s41598-017-15845-0 (PMC5688088; doi:10.1038/s41598-017-15845-0)
Supplement: Supplementary file 1 — Supporting Information [file 41598_2017_15845_MOESM1_ESM.pdf]

Understanding the anticorrosive protective mechanisms of modified epoxy coatings with improved barrier, active and self-healing functionalities: EIS and spectroscopic techniques

Demian I. Njoku[‖†], Miao. M. Cui[‖], Haigang Xiao[‖], Baihui Shang[‖], Ying Li[‖]*

[‖] Laboratory for Corrosion and Protection, Institute of Metal Research, Chinese Academy of Sciences, 62 Wencui Road, Shen Yang, Liao Ning, China, 110016

[†]University of Chinese Academy of Sciences (UCAS), 19A Yuquan Rd, Shijingshan District, Beijing, P. R. China 100049

The corresponding author*

E-mail address: liying@imr.ac.cn

SUPPORTING INFORMATION (SI)

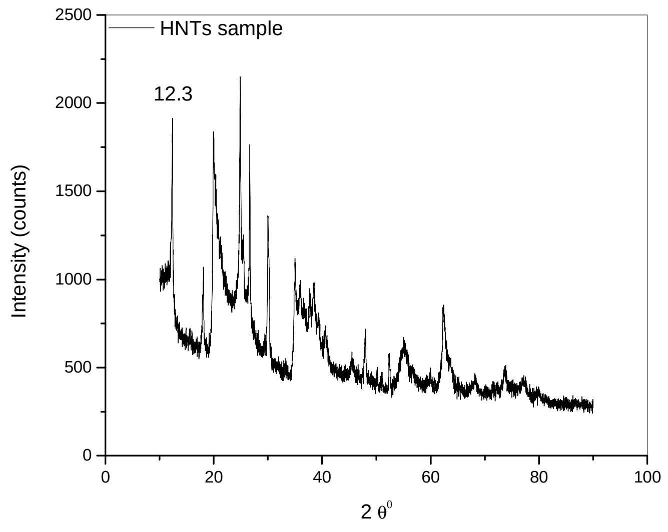

(a)

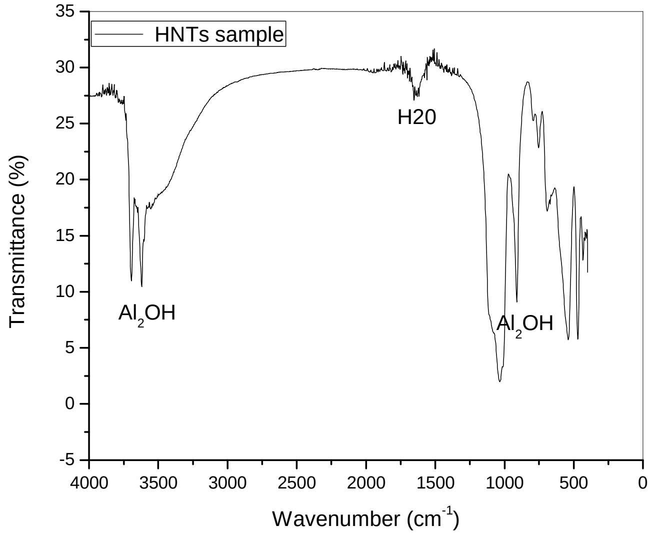

(b)

Fig. SI 1, XRD pattern (a) and FTIR spectrum (b) for the as-received halloysite clay nanotubes

Table SI 1, positions and assignments of the various FTIR absorption peaks for the as-received HNTs clay nanotubes

FTIR Peak Position (cm^{-1})	Assignments	FTIR Peak Position (cm^{-1})	Assignments
3695 and 3620	Al_2–O	755-697	perpendicular Si–O stretching
3618	O–H stretching of inner hydroxyl group	540	deformation of Al–O–Si
1630	OH deformation of water	472	Deformation of Si–O–Si
1025	In-plane Si–O stretching	432	Deformation of Si–O
912	O–H deformation of inner hydroxyl layer		

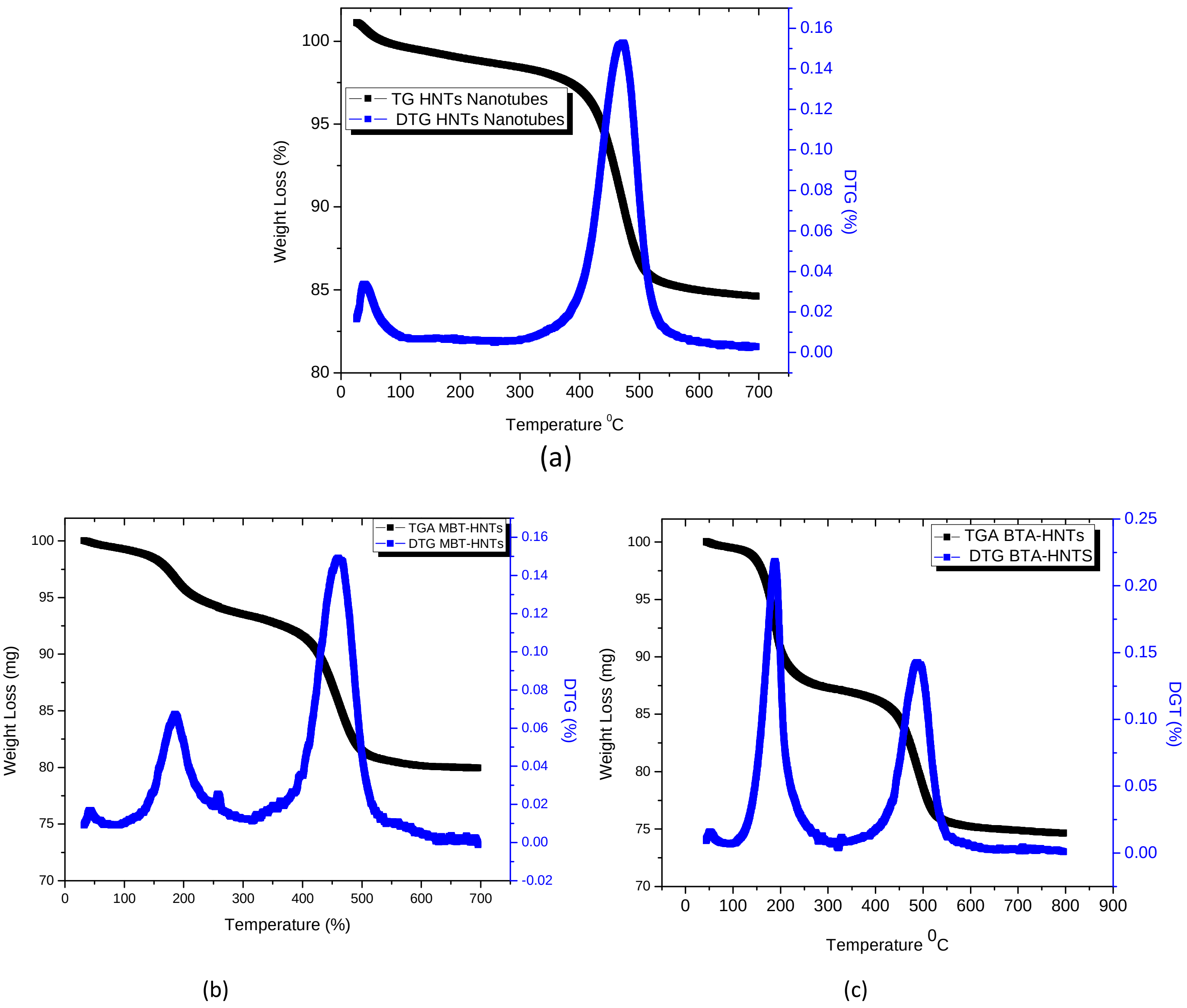

Fig. SI 2, TGA –DTG curves; for HNTs (a) MBT loaded HNTs (b) BTA loaded HNTs (c)

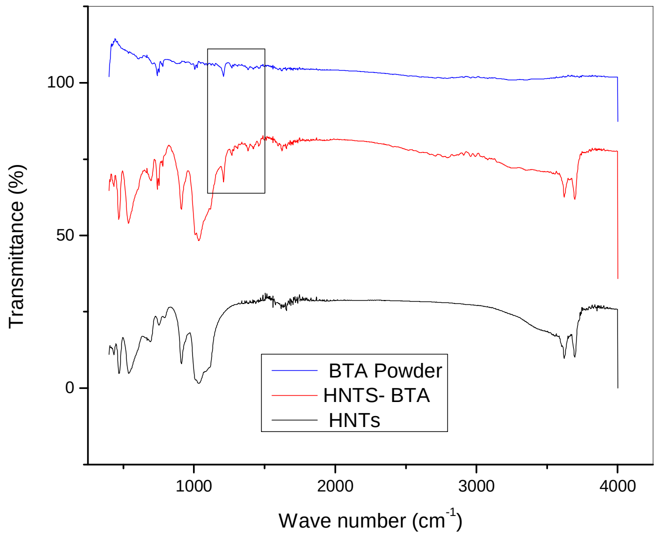

(a)

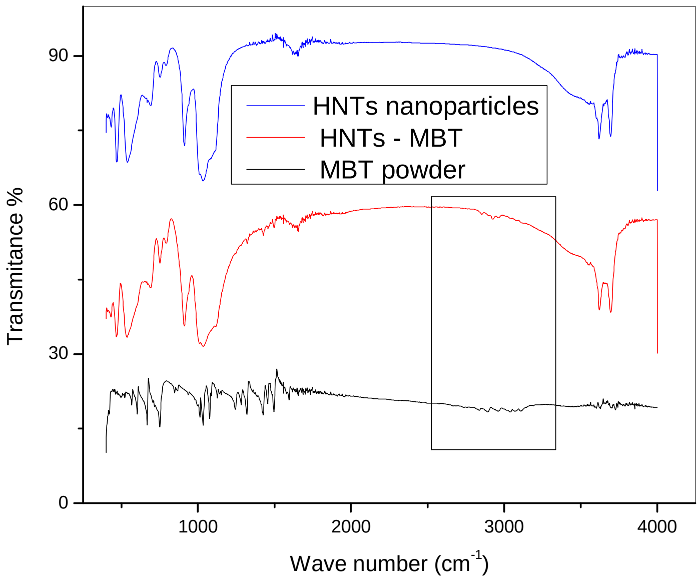

(b)

Fig. SI 3, The FTIR spectra comparing (a) BTA powder, HNTs, and HNTs–BTA (loaded); (b) MBT powder, HNTs, and HNTs–MBT (loaded).

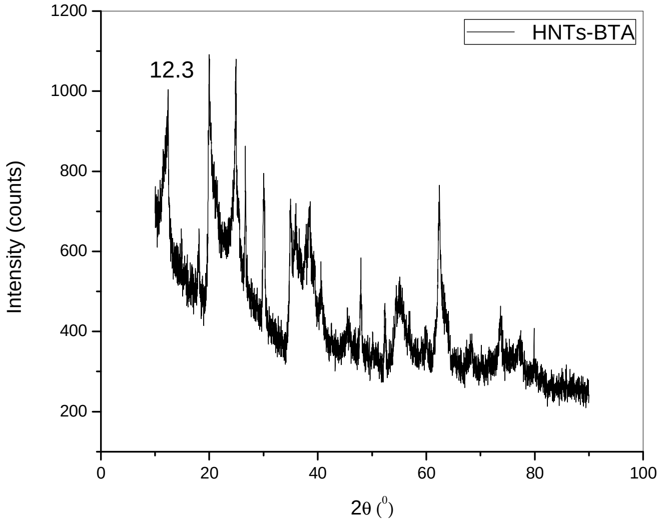

(a)

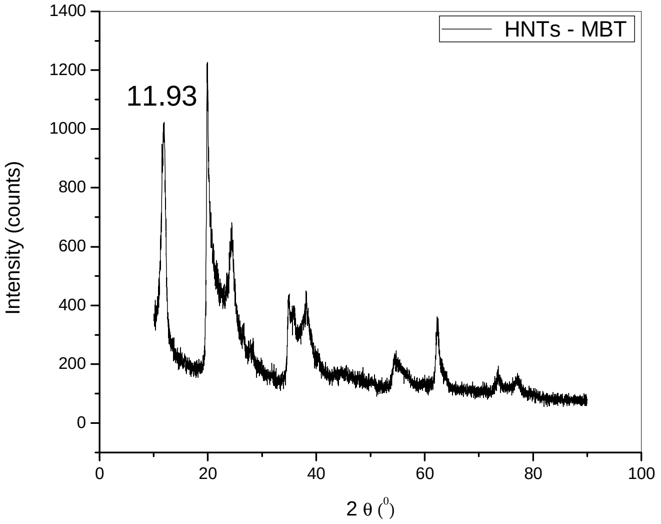

(b)

Fig. SI 4, XRD patterns for HNTs loaded with BTA (a) and MBT (b)

Table SI 2, Constants from the Peppas and Weibull release models

System	K	n	b
BTA	0.53003	0.3657	0.53113
BTA–HNTS	0.1479	0.4829	0.52413
BTA–HNTS –CTS	0.0147	0.6960	0.58878
BTA–HNTs–Fe	0.0128	0.54707	0.5724
MBT	0.6571	0.2012	0.5278
MBT–HNTs	0.4147	0.1557	0.1686
MBT–HNTs–CTS	0.1937	0.2183	0.2327
MBT–HNTs–Fe	0.0588	0.3878	0.3941

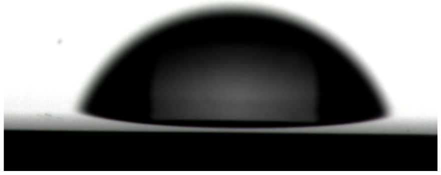

Epoxy (av=64^0)

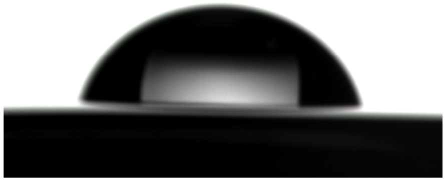

5% HNTs Epoxy Mixture (av=68^0)

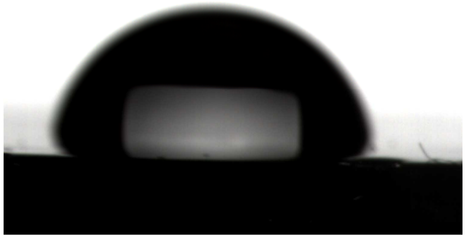

10 % HNTs Epoxy mixture (av=71^0)

Fig. SI 5, Water droplet images and the corresponding average (av) contact angles for epoxy coating and epoxy coating embedded with different amounts of HNTs nanoparticles.

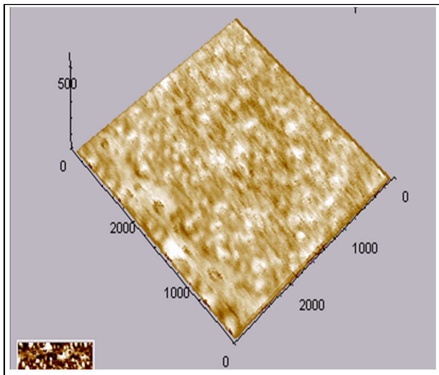

Av. Rf.

13.8 A

Epoxy

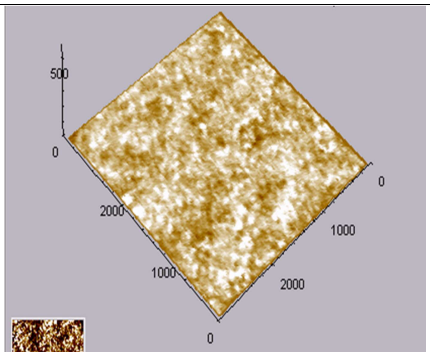

Av. Rf.

15.4 A

Epoxy + 1% HNTs

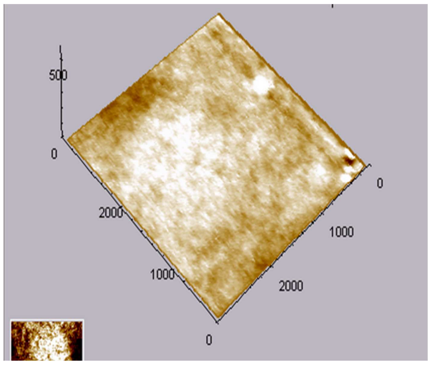

Av.Rf

15.6 A

Epoxy + 5% HNTS

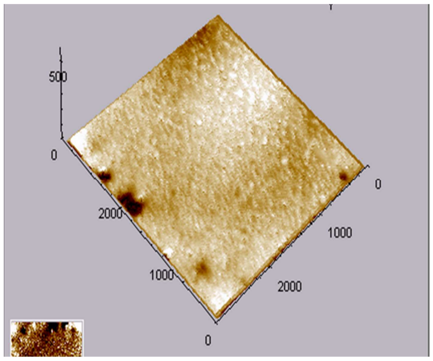

Av.Rf

15.9 A

Epoxy + 10% HNTs

Fig. SI 6, AFM images and the corresponding average surface roughness parameters (Av.Rf) for epoxy coating and epoxy coating embedded with different HNTs particles; (Af = average roughness, A= Armstrong)

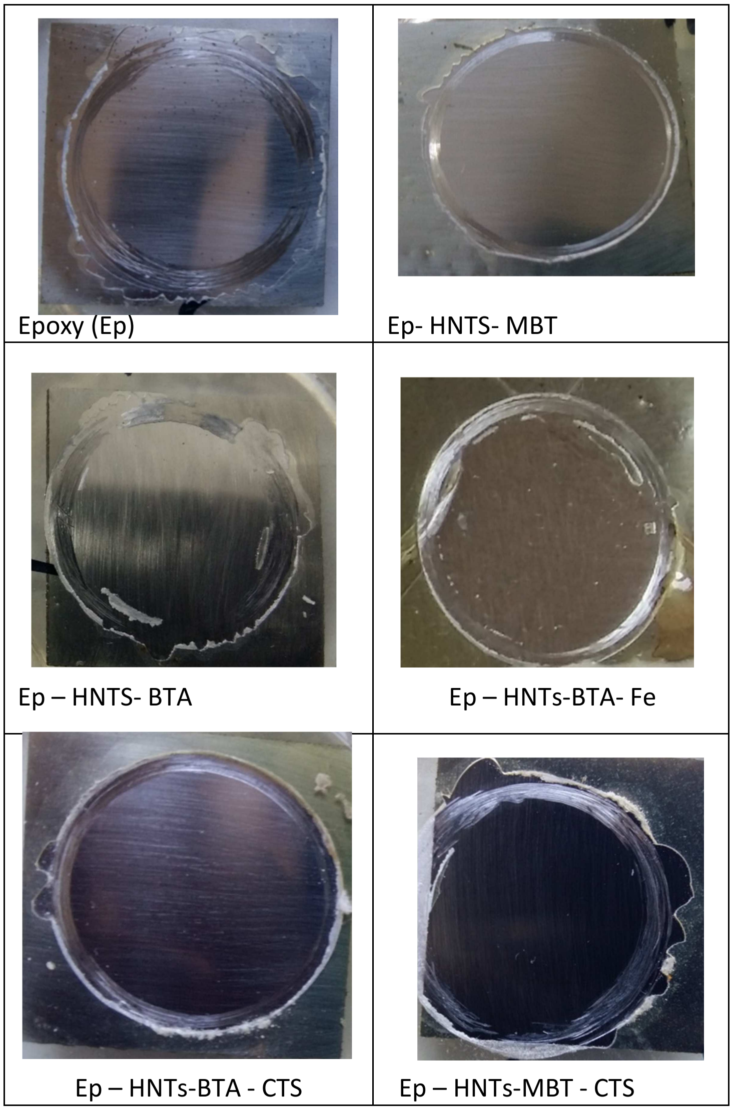

Fig. SI 7, Pictorial (photograph) surface examinations after adhesion test for samples coated with epoxy and the variously modified epoxy coatings immersed in 3.5 % NaCl for 14 days

(a)

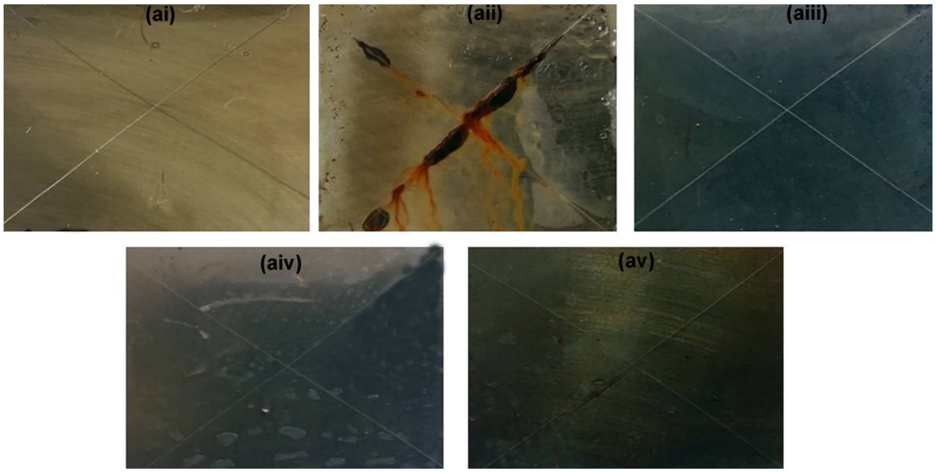

(b)

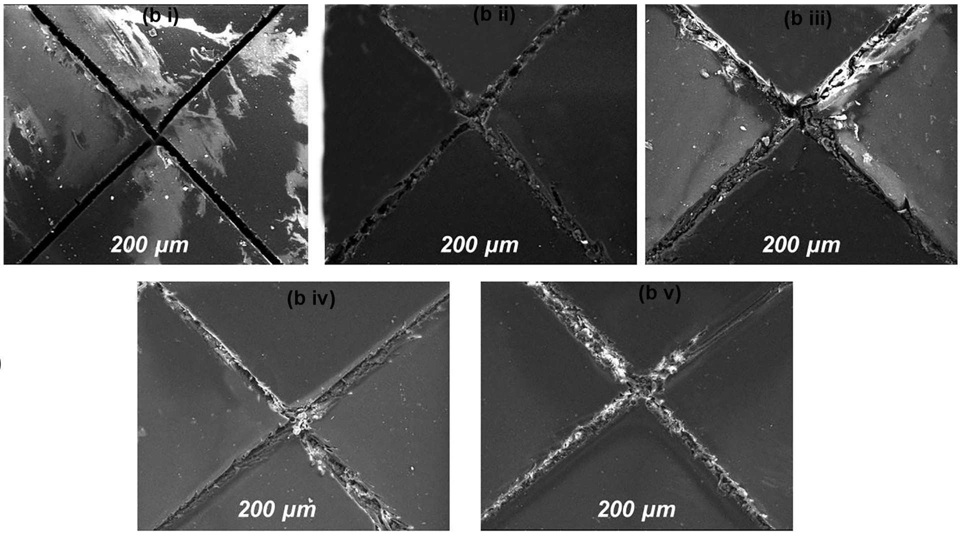

Fig. SI 8a, Photographs comparing the active feedback anticorrosion performances of different epoxy composite coatings applied on Q235 steel panels after 168 h exposure in a salt spray chamber: (a) fresh cut after 0 h, (aii) epoxy reference coating (aiii) epoxy doped with HNTs loaded with BTA (aiv) epoxy doped with HNTs loaded with BTA encapsulated with CTS and (e) epoxy doped with HNTs loaded with BTA capped with Fe. Fig. SI 8b. The corresponding SEM images for fresh cut (bi), and the healing abilities after 24 h immersion in salt solution for epoxy (bii), Ep–BTA (bii), Ep–BTA–CTS (biv) Ep–BTA–Fe (v).

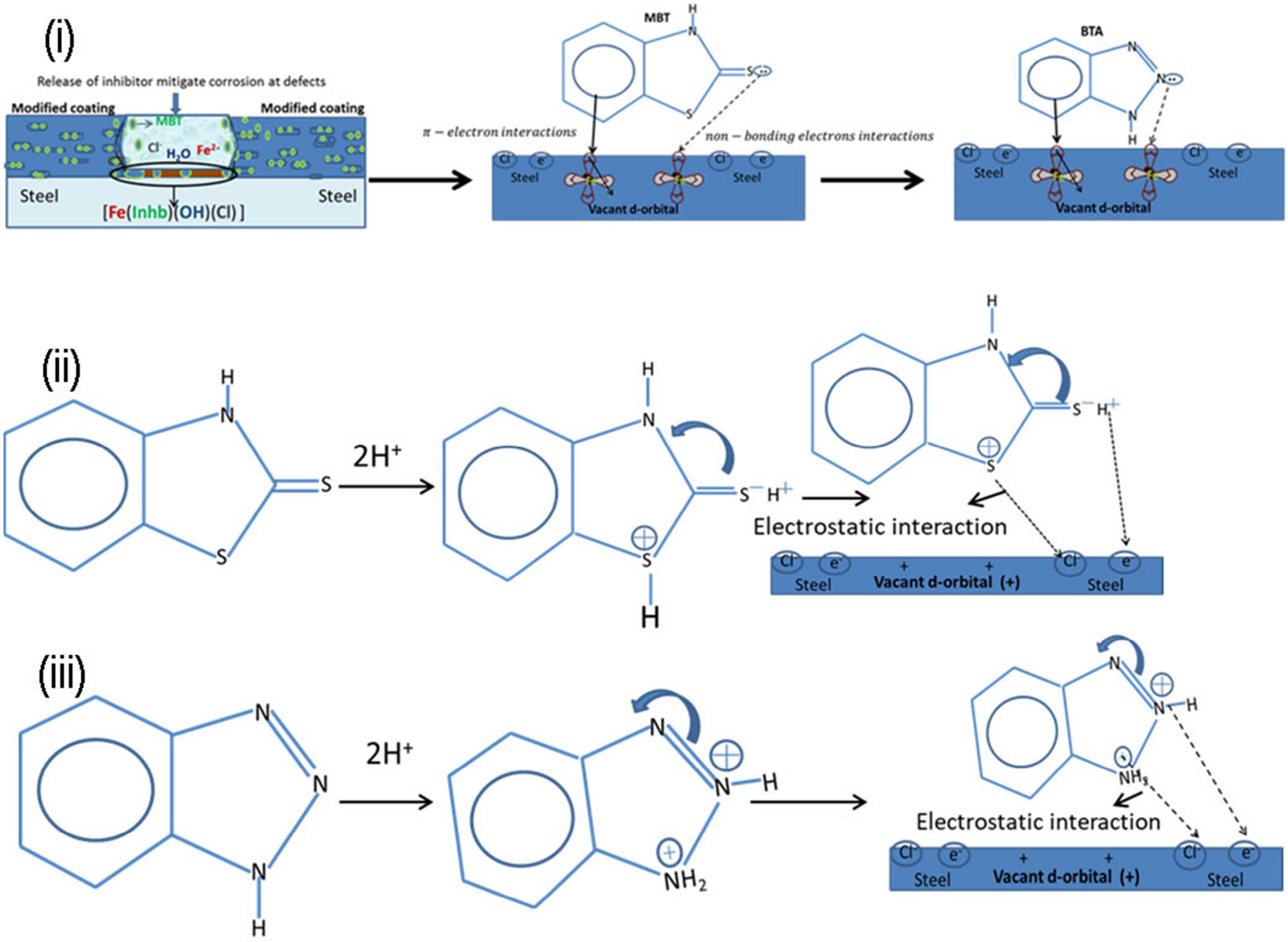

Fig. SI 9, Illustrating (i) the mechanism of active species release from the dispersed HNTs in the epoxy coating and the subsequent adsorption of the released inhibitor molecules on the exposed metal surface; (ii) the protonation mechanism of BTA and (iii) MBT at the anodic sites during corrosion reactions and subsequent adsorption at electrode surface:
(⟶) = pi-electron interaction; (- - - ->) = non-bonding electron interaction; (·········>) = electrostatic attraction.

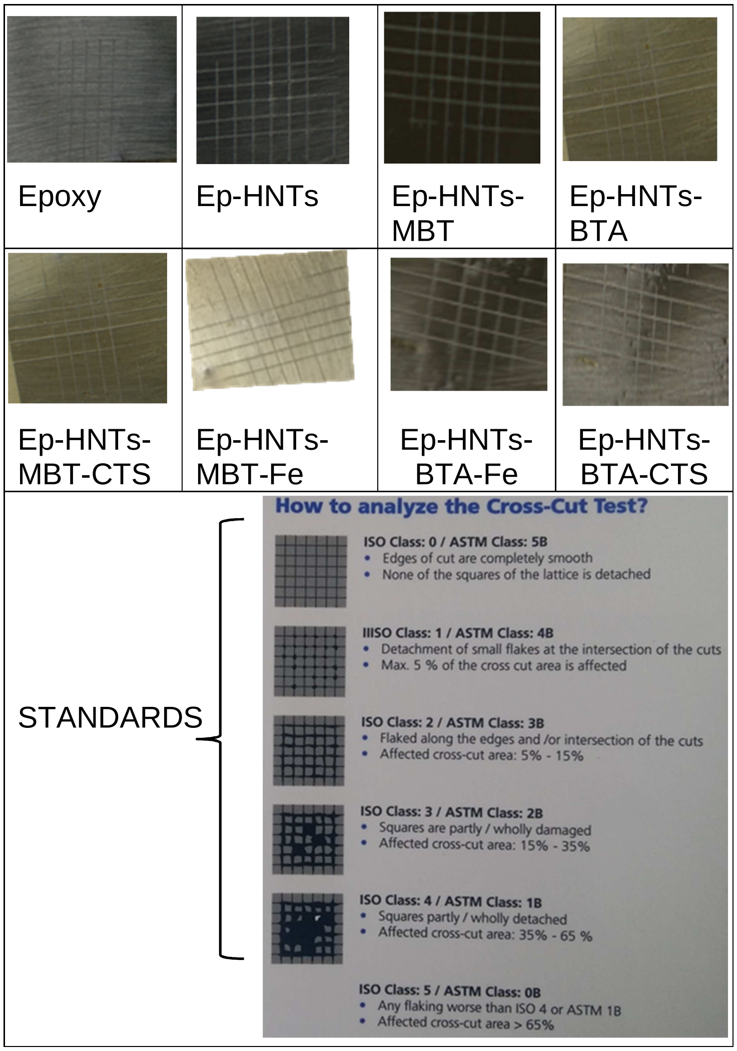

Fig. SI 10, Photographs of the results of the cross-cut adhesion test comparing the squares of the lattice lines on the epoxy coating and the variously modified epoxy composite coatings with the standard.

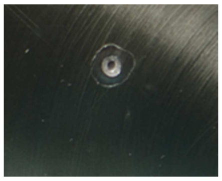 Epoxy (EP)	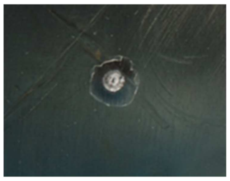 Epoxy (EP)–HNTs
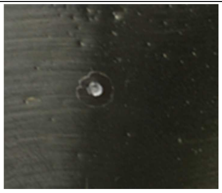 Ep–HNTs–MBT	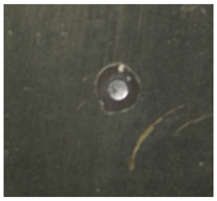 Ep–HNTs–BTA
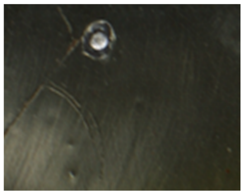 Ep–HNTs–MBT–Fe	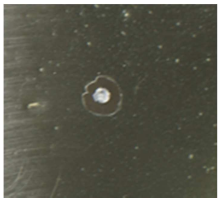 Ep–HNTs–BTA–Fe
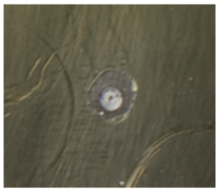 Ep–HNTs–MBT–CTS	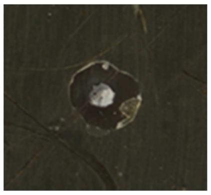 Ep–HNTs–BTA–CTS

Fig. SI 11, Photographs of the various coatings revealing the morphology of the peeled coating areas after the impact test assessments.

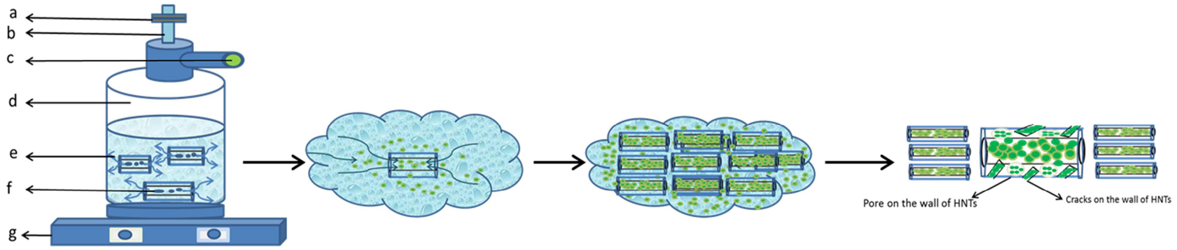

HNTs nanoparticles dispersed in saturated Inhibitor solution

The break of vacuum causes the diffusion of inhibitor molecules into the HNTs lumen and pores

Evaporation under vacuum crystalizes loaded inhibitors in HNTs lumen

Washed, dried and sieved inhibitor loaded HNTs

Fig. SI 12, Illustrating the loading procedure of HNTs with BTA and MBT inhibitors; (a, clip; b, PVC tube; c, pressure pipe to vacuum pump; d, vacuum flask; e, entrapped air bubbles leaving HNTs lumen; f, HNTs dispersed in the inhibitor solution; g, magnetic stirrer).
